# Combustion-related isoprene contributes substantially to the formation of wintertime secondary organic aerosols

**DOI:** 10.1093/nsr/nwae474

**Published:** 2025-01-02

**Authors:** Yanli Zhang, Yatai Men, Hao Guo, Guofeng Shen, Yang Gao, Rui Xiong, Shu Tao, Xinming Wang

**Affiliations:** State Key Laboratory of Organic Geochemistry and Guangdong Key Laboratory of Environmental Protection and Resources Utilization, Guangzhou Institute of Geochemistry, Chinese Academy of Sciences, Guangzhou 510640, China; Laboratory for Earth Surface Processes, College of Urban and Environmental Sciences, Peking University, Beijing 100871, China; State Key Laboratory of Organic Geochemistry and Guangdong Key Laboratory of Environmental Protection and Resources Utilization, Guangzhou Institute of Geochemistry, Chinese Academy of Sciences, Guangzhou 510640, China; Laboratory for Earth Surface Processes, College of Urban and Environmental Sciences, Peking University, Beijing 100871, China; Institute of Carbon Neutrality, Peking University, Beijing 100871, China; Frontiers Science Center for Deep Ocean Multispheres and Earth System/Key Laboratory of Marine Environmental Science and Ecology, Ministry of Education, Ocean University of China, Qingdao 266100, China; Laboratory for Earth Surface Processes, College of Urban and Environmental Sciences, Peking University, Beijing 100871, China; Laboratory for Earth Surface Processes, College of Urban and Environmental Sciences, Peking University, Beijing 100871, China; Institute of Carbon Neutrality, Peking University, Beijing 100871, China; State Key Laboratory of Organic Geochemistry and Guangdong Key Laboratory of Environmental Protection and Resources Utilization, Guangzhou Institute of Geochemistry, Chinese Academy of Sciences, Guangzhou 510640, China

**Keywords:** isoprene, combustion emission, secondary organic aerosol (SOA)

## Abstract

Isoprene is a key reactive organic gas involved in organic aerosol formation. While biogenic isoprene from terrestrial plants has been extensively studied and is recognized as a major contributor to secondary organic aerosol (SOA), high levels of observed SOA, especially in winter, cannot be fully explained by biogenic isoprene alone. In this study, we developed a comprehensive bottom-up emission inventory for isoprene, incorporating both biogenic and combustion sources and modeling their contributions to SOA in China from 2000 to 2016. Combustion-related isoprene emissions from open biomass burning and residential fuel combustion were estimated at 52.0 (39.1–65.7) Gg in 2000, declining to 14.8 (10.6–19.0) Gg by 2016. Open biomass burning contributes ∼40% of combustion-related isoprene emissions. Though, annually, combustion-related isoprene emissions were much smaller than the biogenic emissions, they did account for 32%–80% of total isoprene emissions in many north and west provinces in the colder months in 2016, and were even higher during the early 2000s owing to more biofuel-burning emissions. Model simulation results indicated that combustion-related isoprene could contribute 25%–40% of winter SOA in northern regions. Wintertime isoprene-derived SOA levels declined since 2000, corresponding with decreased combustion-related isoprene emissions; however, the extent of this decline varied regionally due to the influence of other precursors like nitrogen oxides (NOx). In the northeast region with high NOx levels, while combustion-related isoprene emissions decreased by >80% from 2000 to 2016, isoprene-derived SOA declined by only ∼20%. These findings highlight the previously underappreciated contributions of combustion-related isoprene to observed high wintertime isoprene-derived SOA levels.

## Teaser

Isoprene from combustion sources is unignorable, contributing substantially to the formation of wintertime secondary organic aerosol, despite its lower annual emissions compared to the biogenic isoprene.

## INTRODUCTION

Atmospheric aerosol is associated with adverse health effects, reduced visibility and climate change [1]. Ambient organic aerosols (OAs) include primary organic aerosol (POA), which is directly emitted from sources like fuel combustion, and secondary organic aerosol (SOA), which is formed through the atmospheric oxidation of gaseous precursors. Globally, SOA contributes ∼30%–90% of the total OA mass, varying in region and period [2]. SOA formed by the oxidation of volatile organic compounds (VOCs) emitted from human activities (anthropogenic) and vegetation (biogenic) significantly contributes (20%–80%) to aerosol levels with notable seasonal and spatial variations [3]. Isoprene (2-methyl-1,3-butadiene, C_5_H_8_), as a biogenic VOC (BVOC) primarily emitted from broad-leaf trees [4], exhibits substantially greater global atmospheric emissions compared to other human-made or natural VOCs [5]. As a highly reactive organic gas, isoprene's role as a precursor to SOA has been corroborated by both field observations [6–8] and laboratory studies [9–13], while model assessments identify it as the largest contributor to SOA among volatile/semi-volatile organic compounds (SVOCs) [3]. Consequently, uncertainties surrounding isoprene emissions and their atmospheric behavior could impede accurate estimation of isoprene-derived SOA.

Biogenic isoprene emission models, such as the Model for Emissions of Gases and Aerosols from Nature (MEGAN) [9], have long been developed to estimate isoprene emissions from terrestrial ecosystems [14–16]. They are widely incorporated into atmospheric chemical transport models (CTMs) or earth system models to assess the impact of biogenic isoprene emission on air quality and climate [17]. Regarding non-biogenic isoprene emissions, while extensive field measurements have shown that biomass burning can release a significant amount of isoprene [18,19], these emissions are still often disregarded in emission inventories and assessments of atmospheric influences. Studies have identified other anthropogenic sources of isoprene beyond biomass burning, including human exhalation and microbial emission [20–22], and even vehicular exhaust [23,24]. However, these emissions are expected to contribute minimally on global or regional scales. While some earlier studies observed isoprene in vehicle exhaust [23,24], recent tunnel studies report no significant isoprene emissions from vehicles [25–27].

The absence of non-biogenic isoprene emissions in the model has led to an obvious underestimation of SOA in the outputs. For example, Karl *et al.* (2009) [28] reported a substantial underestimation of organic matter (OM) in Europe, likely due to the omission of non-methane VOCs (NMVOCs) from wood burning in the wintertime emission inventory. Similarly, Murphy *et al.* (2017) [29] demonstrated that the inclusion of potential anthropogenic combustion NMVOCs in CTMs could reduce the SOA modeling bias from 1.14 μg m^−3^ to 0.73 μg m^−3^ in the USA during winter 2011. Although these studies addressed combustion-related NMVOCs broadly rather than isoprene specifically, isoprene remains an important component of NMVOCs in SOA formation. One-year observations at 12 sites across China demonstrated unexpected increases in particulate isoprene-SOA tracers during winter, when biogenic isoprene emissions are typically negligible [30]. These increases were found to be significantly correlated with the biomass-burning tracer levoglucosan [30], indicating that elevated combustion emissions contribute substantially to wintertime isoprene-derived SOA levels. Abnormally high levels of isoprene in ambient air were observed at some sites in northern China during winter [31], with synchronized enhancements observed alongside combustion tracers. These observations highlight the importance of considering non-biogenic sources of isoprene and their potential atmospheric effects, indicating a need for a comprehensive emission inventory.

To quantitatively assess the impacts of non-biogenic isoprene from combustion sources and improve the performance of current CTMs in predicting isoprene-derived SOA, we have developed a high-resolution (36 × 36 km^2^) combustion isoprene emission inventory, and modeled its contribution to ambient SOA in all four seasons from 2000 to 2016 in China (see Methods section). Our results indicate that isoprene-derived SOA accounts for 25%–40% of the total SOA during winter. Incorporating combustion source isoprene significantly enhances the accuracy of model predictions for both isoprene mixing ratios and isoprene-derived SOA.

## RESULTS AND DISCUSSION

### Combustion-related and biogenic isoprene emission estimates

Through the integration of both biogenic and combustion emissions (see Methods section), the total isoprene emission in China was 5.9 Tg in 2000, but 12.6 Tg by 2016 (Table S1), predominantly from the biogenic emissions on the national scale. The biogenic isoprene emissions were ∼12.6 Tg in 2016, which is comparable to the estimates of 13.3–14.3 Tg during the period 2015–2019 in previous studies [32,33]. The combustion-related isoprene emissions were only 14.8 (10.6–19.0) Gg in 2016 (Fig. S1), and 52.0 (39.1–65.7) Gg in 2000. This declining trend was closely associated with the transition to cleaner household energies, namely from traditional biofuels to clean energies like gas and electricity [34], since residential combustion is one major source of combustion-related isoprene (Figs S2 and S3).

**Figure 1.**
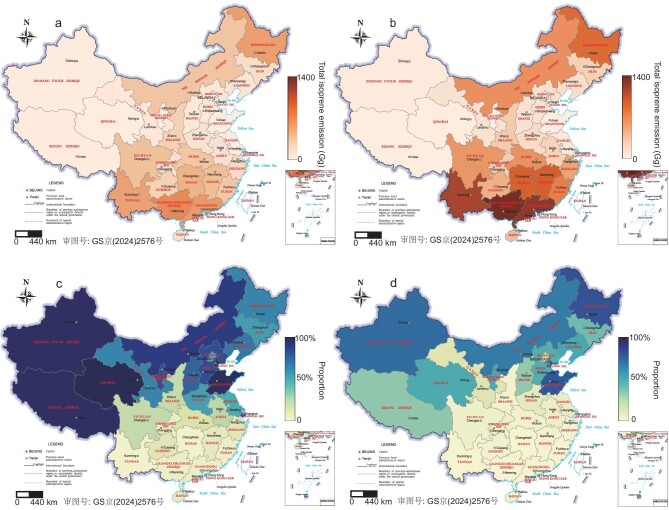
Spatial distribution of the total annual isoprene emissions in (a) 2000 and (b) 2016; and the proportion of combustion-related isoprene emission to the total from both biogenic and combustion sources in February of (c) 2000 and (d) 2016.

Isoprene emissions had a notable spatial difference, as shown in Fig. 1. For biogenic sources, high isoprene emissions are primarily located in the south and northeast region, particularly in areas with extensive broad-leaved forests, while the western Qinghai-Xizang Plateau and the northwest area have much lower biogenic emissions (Fig. 1). Such spatial distribution is nearly unchanged over the 20 years that were studied (Fig. S4). However, for the combustion-related isoprene, the spatial distribution is very different, and moreover, the pattern did change over the study period (Fig. S4). In the early 2000s, combustion-related isoprene emissions were more concentrated in the west, mainly due to extensive biofuel burning in the residential sector. With the promotion of cleaner household energies [35], emissions from open biomass burning became notably significant; areas like the northeast region have relatively higher combustion-related isoprene emissions now (Fig. S5).

The newly developed inventory provides detailed monthly isoprene emissions. The spatiotemporal analysis highlights notable seasonal variations in emission amounts and sources, with substantial contributions of combustion-related isoprene in several regions, particularly during the cold winter months (Figs S6 and S7). In 2000, combustion-related isoprene emissions in winter accounted for ∼40%, and varied between 10% and 45% of the annual total combustion-related isoprene emissions in different provinces (Fig. S8). By 2016, despite reduced emissions during the study period, wintertime emissions were still ∼30% of the annual total. This percentage was relatively low in the southeast coastal areas (15%–20%) and higher in the colder northern regions (40%–45%). High wintertime emissions of combustion-related isoprene and its spatial difference were closely associated with the residential emissions, in which more biofuels were used for space heating in cold periods, especially in the western and northern areas [36,37].

Significant temporal differences exist in not only the absolute emission amounts of combustion-related isoprene, but in its relative contribution to the total isoprene from both biogenic and combustion sources, as both biogenic and combustion-related isoprene had distinct monthly changes. Monthly emissions and relative shares of combustion-related isoprene to the total are illustrated in Fig. S9, and Fig. 1 compares the relative share of combustion-related isoprene to the total in 2000 (Fig. 1c) and 2016 (Fig. 1d) in February, as an example of a cold winter period. As mentioned above, annually the combustion-related isoprene is small compared to that from biogenic sources (Fig. S1), however, the relative contribution is unignorable in winter, especially in north and west regions. In February 2016, the combustion-related isoprene contributed ∼32%–80% of total isoprene emissions in many north provinces. In 2000, the relative contribution was 53%–98% in those areas, and has since declined due to reduced biofuel use as part of China's clean energy transition.

### Seasonal SOA production from biogenic and combustion-related isoprene

**Figure 2.**
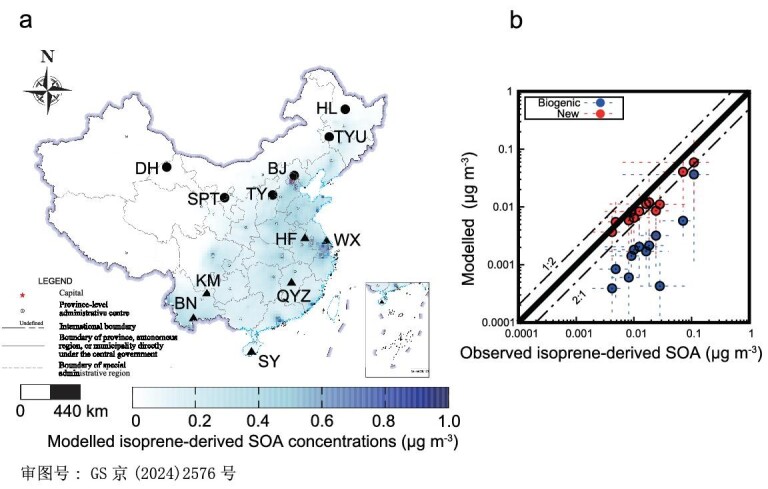
(a) Observation-site map of isoprene-derived SOA (winter 2013 in Ding *et al.* [30]). The color code represents the modeled isoprene-derived SOA concentrations in the New inventory in winter 2013. (b) Comparison of observed and modeled isoprene-derived SOA in Biogenic and New (Biogenic + Combustion) inventories at 12 sites. The Biogenic and New inventories consider biogenic isoprene emissions and combustion-related isoprene emissions, respectively.

To quantify the SOA formation from combustion-related isoprene, two emission inventories were incorporated into the Weather Research & Forecasting (WRF)/Community Multiscale Air Quality (CMAQ) modeling system: one without combustion-related emissions (Biogenic) and another including them (New: Biogenic + Combustion), as described in the Methods section. Despite inherent uncertainties in SOA modeling and potential discrepancies between modeled and observed values [38,39], the utilization of the two contrasting emission inventories, alongside consistent meteorology and chemical mechanisms within the model, effectively elucidates the impact of combustion-related isoprene. Compared with isoprene-derived SOA observations at 12 sites across China in winter 2013 [30] in Fig. 2a, excluding combustion-related isoprene led to a significant underestimation of isoprene-derived SOA, with a difference of up to 66-fold in some cases. As seen in Fig. 2b, after including combustion-related isoprene in the model, the SOA predictions were much closer to the observed values, with a relative difference reduced within 2-fold. Additionally, the inclusion of combustion isoprene improved the spatial representation of isoprene-derived SOA in the model in Fig. 2b. Ding *et al.* (2016) [30] measured isoprene-derived SOA tracers, including 3-methyl-2,3,4-trihydroxy-1-butene, 2-methylglyceric acid, 2-methylthreitol and 2-methylerythritol. With concentrations of these tracers, they estimated isoprene-derived SOA using the SOA-tracer method developed by Kleindienst *et al.* (2007) [40], which assumes that the mass ratios of these tracers to SOA in ambient air are the same as those observed in chamber studies simulating SOA formation from isoprene. Ding *et al.* (2016) [30] reported an overall uncertainty of ∼47% for estimated isoprene-derived SOA, based on error propagation. Despite this uncertainty, the observation of isoprene-derived SOA cannot explain the much lower SOA levels predicted by the model, compared to those observed in the field.

**Figure 3.**
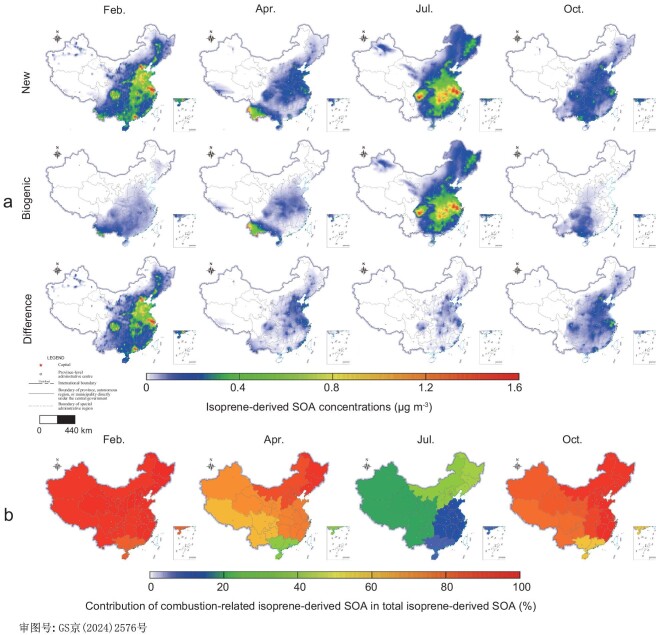
(a) Seasonal variations of isoprene-derived SOA (μg/m^3^) in the Biogenic and New inventories and their differences in 2016. (b) Contributions of combustion-related isoprene-derived SOA in total isoprene-derived SOA (%) over seven major regions in China in 2016.

Seasonal variations of isoprene-derived SOA in both Biogenic and New inventories in 2016 are illustrated in Fig. 3a, highlighting the significance of combustion-related isoprene emissions in winter SOA production. In the Biogenic inventory, where only biogenic isoprene emissions are considered, isoprene-derived SOA concentrations were lower in winter (<0.2 μg/m^3^) but markedly higher in summer (∼1.4 μg/m^3^), particularly in the eastern regions and the Sichuan Basin.

In the New inventory, which incorporates both biogenic and combustion emissions, the model's simulation of surface isoprene mixing ratios was much closer to the observations compared to previous simulations, which lacked combustion-related isoprene sources [31] (see discussion in Methods). Notably, the modeled isoprene-derived SOA in winter was corrected in the New inventory compared to the Biogenic inventory (Fig. S10), with an average increase of 0.4 μg/m^3^ nationwide and up to 1.5 μg/m^3^ in regions such as northeastern China and the Sichuan Basin. In summer, however, the differences were negligible (<0.1 μg/m^3^). The modeled combustion-related isoprene contributed more than 80% in total isoprene-derived SOA nationwide in winter (Fig. 3b), but less than 40% in summer.

In the Biogenic inventory, isoprene-derived SOA could account for up to 50% of the total SOA in summer across China, while its contribution was insignificant in other seasons and clearly biased (up to 0.5 μg/m^3^). In the New inventory (Fig. S11), SOA from biogenic isoprene contributed 20%–40% in summer and <10% in winter to the total SOA. In contrast, SOA from combustion-related isoprene contributed 5%–20% in summer and 25%–40% in winter to the total SOA. The proportion of isoprene-derived SOA in total SOA in winter was corrected, and increased by 10%–20%, accounting for over 50% in some northern and eastern provinces in the New inventory, while other VOCs/SVOCs still contributed to SOA formation and accounted for ∼50% of the total SOA in winter. Therefore, the corrected modeled isoprene-derived SOA in the New inventory underscores the importance of considering combustion sources, especially in winter.

### Regional differences in historical SOA changes in response to declined isoprene emissions

In contrast to the nationwide increase in biogenic isoprene emissions, driven by the expansion of green spaces and rising temperature, the quantities and proportions of combustion-related isoprene emissions within the national total showed a notable decline (Fig. S1). Across China, with the exception of Chongqing and Heilongjiang provinces, annual combustion-related isoprene emissions consistently showed a downward trajectory, with decline rates ranging from ∼3% to 95% in different provinces between 2000 and 2016 (Fig. S12). Despite the reduction in combustion-related isoprene emissions, it is worth noting that isoprene from combustion sources continued to contribute significantly to the formation of SOA, especially in certain northern regions in China during winter.

**Figure 4.**
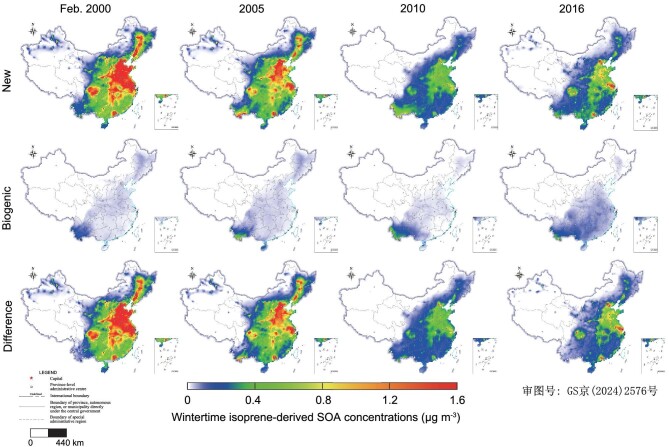
Historical changes in wintertime isoprene-derived SOA concentrations (μg/m^3^) in the Biogenic inventory and New inventory and their differences.

Corresponding with changes in isoprene emissions, isoprene-derived SOA, both in terms of mass concentrations and relative contributions, is expected to evolve over time. As depicted in Fig. 4, with the decrease in combustion-related emissions, total isoprene-derived SOA concentrations declined, while SOA from biogenic isoprene exhibited a steady increase. The proportions of isoprene-derived SOA in total SOA during winter are also presented in Fig. S13. In the Biogenic inventory, the contribution of isoprene-derived SOA increased from 2000 to 2016 due to rising biogenic isoprene emissions, particularly noticeable in southern China. Nevertheless, isoprene-derived SOA still accounted for <10% of the total SOA during winter. In the New inventory, the proportions of SOA from combustion-related emissions in the total SOA decreased from 2000 to 2016 on average (national), albeit with varying rates among regions (Fig. S14).

From 2000 to 2016, nationwide wintertime SOA from combustion-related isoprene decreased from 3.0 μg/m^3^ (with a range of 1.2–3.9 μg/m^3^) in 2000 to 1.2 μg/m^3^ (with a range of 0.4–1.6 μg/m^3^) in 2016, with declining rates varying across regions (Fig. S15). Specifically, wintertime SOA formation from combustion-related isoprene decreased by ∼75% in North China and 50% in South China during the same period.

**Figure 5.**
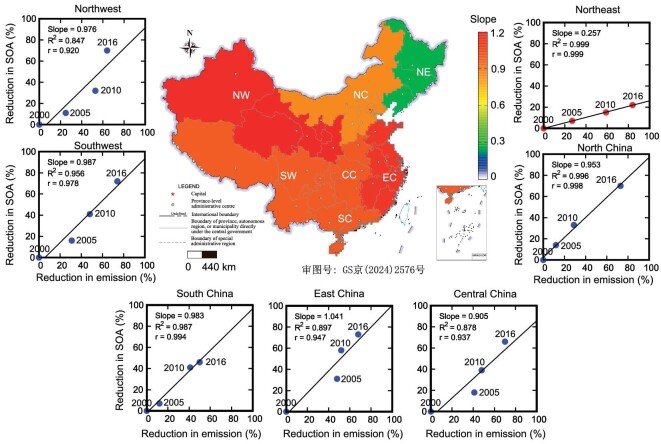
The slopes of reduction in isoprene-derived SOA (served as x) and reduction in combustion-related isoprene emissions (served as y) in seven major regions of China. The color code represents the average slope of each region.

Interestingly, the decline in SOA formation from combustion-related isoprene appeared to be disproportionate to the decrease in combustion-related isoprene emissions (Fig. 5), and this disproportionality also exhibited regional variations. For instance, in northeastern China, while combustion-related isoprene emissions decreased by over 80% from 2000 to 2016, the SOA produced from combustion-related isoprene only decreased by ∼20%. These varied decreasing slopes, as shown in Fig. 5 across different regions, reflected the complexity of SOA formation processes involving diverse precursors [3,41,42]. Although the mechanisms governing SOA formation from isoprene are not yet fully understood, the level of nitrogen oxides (NOx) plays a crucial role in RO_2_ chemistry, which is integral to SOA formation as described in the CMAQ model [3,41]. Regarding combustion-related isoprene, differences in the relationship between SOA and emissions may stem from distinct spatial-temporal patterns of NOx in different regions and the intricate interplay between isoprene and NOx in SOA formation.

**Figure 6.**
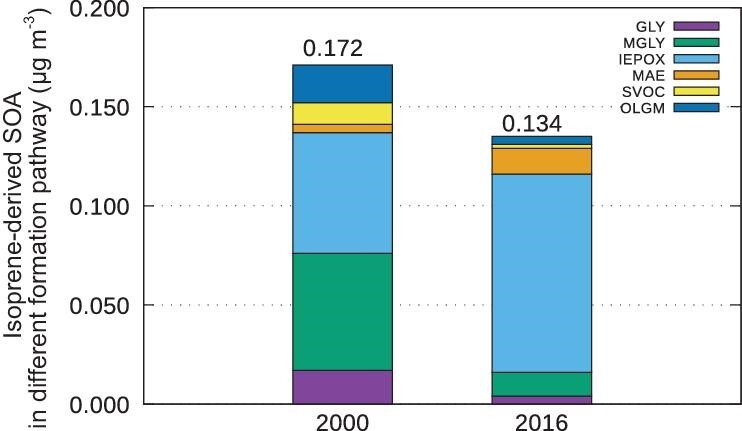
Comparison of isoprene-derived SOA formation pathways in wintertime, Northeast China in 2000 and 2016 [glyoxal SOA (GLY), methylglyoxal SOA (MGLY), isoprene epoxydiol SOA (IEPOX), methacrylic acid epoxide SOA (MAE), semivolatile organic compound SOA (SVOC) and oligomers (OLGM) as described in Ying *et al.*, 2015 [43]].

To investigate the discrepancy between the substantial decrease in isoprene emissions and the relatively minor decrease in isoprene-derived SOA concentrations in northeast China during winter, Fig. 6 was created to illustrate the significant changes in major formation pathways between 2000 and 2016. In 2000, isoprene oxidation products glyoxal (GLY) and methylglyoxal (MGLY) collectively contributed >40% to isoprene-derived SOA, with isoprene epoxydiol (IEPOX) also accounting for >30%. However, by 2016, the contributions of ‘GLY + MGLY’ decreased to 12%, while the contribution of IEPOX surged to 74%, nearly dominating the formation processes. As discussed in Ying *et al.* (2015) [43], the decline in the ‘GLY + MGLY’ formation pathway and the increase in the IEPOX pathway were anticipated with reductions in anthropogenic NOx emissions. Specifically, NOx emissions in the Multi-resolution Emission Inventory for China (MEIC) inventory decreased by 28% during this period, which may explain the distinct pattern observed in Northeast China compared to other regions. Additionally, Lin *et al.* (2013) reported that aerosol surface uptake of IEPOX formed from isoprene photo-oxidation under low-NOx conditions [44]. Overall, the reduction of NOx levels in Northeast China from 2000 to 2016 likely contributed to a shift in the major isoprene-derived SOA formation pathway, leading to increased SOA productivity.

### Implications

Isoprene is a highly reactive organic gas with global emissions vastly surpassing that of any other VOCs, and isoprene-derived SOA accounts for ∼70% of the global total SOA [3]. Therefore, accurate estimates of isoprene emissions are crucial for understanding atmospheric SOA chemistry. In this study, we extended our focus on biogenic emissions to include combustion emissions, specifically combustion sources, and compiled high-resolution isoprene emission inventories for China from 2000 to 2016. The updated emission inventories significantly improved the model's performance in predicting levels of wintertime isoprene and isoprene-derived SOA. This explains the unusually high levels of isoprene and isoprene-derived SOA tracer observed in certain parts of China during winter [30,31].

Our findings reveal that combustion-related isoprene plays a minor role in contributing to SOA during summer compared to biogenic isoprene. However, during winter, when severe aerosol pollution events occur more frequently, combustion-related isoprene could contribute up to 40% of total SOA. This highlights the importance of including combustion sources in isoprene emissions to comprehensively address its role in air chemistry and pollution.

Furthermore, our results demonstrate a drastic decrease in combustion-related isoprene emissions in China from 2000 to 2016, largely due to the transition from solid fuels to cleaner energy sources. This reaffirms the co-benefits of energy transition in reducing reactive organic gases such as isoprene for better air quality, especially in less developed regions where solid fuels are still prevalent [45]. Moreover, combustion sources of isoprene may play a more significant role in regions such as Southeast Asia, South America and Africa, where residential solid fuel use and wildfires are prevalent [46]. This study highlights the need for future research to be done on a global scale, enhancing our understanding of combustion-related isoprene emissions and informing better air quality strategies.

Despite the significant decrease in combustion-related isoprene emissions during 2000–2016, modeled isoprene-derived SOA in winter decreased to a lesser extent, suggesting that factors other than emissions also influence the role of isoprene in SOA formation. Although NOx is considered to be among these factors, the non-linearity of this relationship is not fully understood.

It is important to note that the CTM simulation in this study updates the isoprene emissions and enhances the SOA formation chemistry to incorporate detailed isoprene oxidation pathways [43]. While the study did significantly improve modeled wintertime isoprene-derived SOA levels by including combustion-related emissions, the model results still substantially underestimate observed levels. SOA formation pathways are not yet fully understood or accurately represented in current models. For example, due to limited knowledge of highly oxygenated compounds’ condensed-phase or heterogeneous reactions, accurately representing this chemistry in the model remains a challenge [47]. Advancing scientific understanding of isoprene chemistry in SOA formation processes will be essential for improving atmospheric models and reducing model uncertainty. However, uncertainties remain due to the lack of long-term isoprene and isoprene-derived SOA observations to validate modeling trends, also, the uncertainties rooted in the emission estimates for combustion-related isoprene sources, especially as emissions from wildfires continue to increase, influenced by smoke–weather interactions within the context of global climate change [48]. Additionally, relatively abundant isoprene SOA tracers are widely observed over oceans [49,50], potentially linked to wildfires [49,51] or sea–air interfacial photochemistry [52]. Despite these uncertainties, recognizing combustion-related isoprene emissions helps to narrow the knowledge gap between observed wintertime SOA levels and model predictions. Furthermore, the decrease in combustion-related isoprene emissions evidences the co-benefits of energy transition efforts in China, as reduction in reactive organic gases like isoprene contribute to improved air quality.

## METHODS

### Emission inventory compilation

The bottom-up inventory was compiled by summing the products of combustion activities and their corresponding emission factors (EFs). These activities, included in the isoprene emission inventory for combustion sources, encompassed a range of sources such as forest wildfires, outdoor combustion of agricultural waste and residential fuel combustion.

The MEICv1.3 developed by Tsinghua University (http://www.meicmodel.org) provides total NMVOC emissions, but it does not specifically account for combustion-related or biogenic isoprene. Typically, modeling studies estimate anthropogenic isoprene emissions by applying a fraction factor in total NMVOCs to isoprene, which obviously introduces large uncertainties. Here, a bottom-up isoprene emissions inventory was developed by compiling available EF data from literature studies (Text S1 and Table S3) and fuel consumption data from the Global Emission Modeling System (GEMS, formerly PKU-FUEL, https://gems.pku.edu.cn). Residential fuel consumption data in the GEMS offer county-level fuel consumption information for China with monthly time resolution. Research findings suggest that this inventory provides more precise estimates of emissions from residential sources in China compared to other inventories like Emissions Database for Global Atmospheric Research (EDGAR) and Community Emissions Data System (CEDS) [53,54]. For data on forest wildfires and open-air burning of agricultural waste, the Global Fire Emission Database version 4.1s (GFED4.1s) was adopted. GFED4.1s offers global estimates of monthly burned areas at a resolution of 0.25° × 0.25° for different fire types [55] and is widely employed in studies related to fire emissions [56,57]. To ensure consistency in spatial resolution, the results obtained from both inventories were aggregated and standardized at the provincial level.

While the industrial and power sectors do utilize fuels capable of emitting isoprene, such as biomass pellets, it is important to highlight that factories commonly employ flue gas treatment measures to reduce emissions. Isoprene is typically treated before being discharged; however, research concerning the efficiency of isoprene treatment in industrial sectors is currently scarce. As a result, this study did not specifically delve into isoprene emissions from the industrial and power sectors due to the limited information available regarding the effectiveness of their treatment methods. Also, as mentioned above, isoprene emissions from human exhalation and vehicular exhaust are not considered here. Our combustion-related isoprene inventory covers isoprene emissions from open biomass burning (including forest fires and agricultural waste burning) and residential biomass combustion. These sources are categorized into four groups, as outlined in Table S2. The inventory spans from 2000 to 2016, with monthly temporal resolution and provincial spatial resolution. EFs for various sources were collected via a comprehensive literature review (Table S3). This bottom-up inventory of combustion-related isoprene improves the accuracy of isoprene emission estimates by reducing the uncertainty associated with applying a constant but small fraction factor in anthropogenic NMVOCs to isoprene.

### Uncertainty analysis

Monte Carlo simulations were performed to evaluate the uncertainties within the compiled inventory arising from combustion activity and EF data. The simulation was iterated 10 000 times, randomly sampling combustion activity intensity and EF data from diverse sources. The outcomes were presented as medians, upper quartiles and lower quartiles derived from Monte Carlo simulation. Python (Version 3.10.7) was utilized for both the Monte Carlo simulation and data analysis, with virtual environments managed through Anaconda.

### Model description

The CMAQ v5.0.2 model system developed by the US Environmental Protection Agency Atmospheric Science Modeling Division was used in this study. The original model underwent modifications to incorporate a more detailed SAPRC-11 photochemical mechanism, enhancing the representation of isoprene oxidation chemistry pathways. As this study focuses specifically on isoprene-derived SOA, the modeling process tracked the various photo-oxidation pathways of isoprene that lead to SOA formation. These modifications enabled the model to simulate the formation of SOA from surface-controlled reactive uptake of dicarbonyls, IEPOX and methacrylic acid epoxide (MAE) [43], as well as in-cloud processing of isoprene oxidation products GLY and MGLY [58,59]. In this study, the S11 chemical mechanism, which includes a standard group of VOCs, was modified to incorporate more detailed isoprene oxidation chemistry. This includes the expanded formation pathways for IEPOX, a more detailed representation of isoprene nitrate chemistry, and the inclusion of reactions describing the formation of MAE and hydroxymethyl-methyl-α-lactone (HMML) from the oxidation of methacyloyl-peroxy nitrate (MPAN). The modified S11 mechanism was further enhanced to track GLY and MGLY formation from major precursor groups, using precursor-tagged species. For instance, GLY_I represent GLY formed from isoprene. Additionally, oxidation products leading to GLY and MGLY formation from different precursor groups were tagged to identify their origin. For example, acetaldehyde (CCHO), which can be oxidized to form glyoxal, is represented as CCHO_I1 when derived from isoprene. This tagging allows us to trace the precursor origins of second- and later-generation oxidation products of GLY and MGLY. A conceptual figure illustrating the isoprene-derived SOA formation pathways is provided in Fig. S17. Further details regarding the modified mechanism and model can be found in Ying *et al.* [43]. The improved CMAQ model has undergone testing and validation for analyzing SOA formation in various regions, including Eastern US [43], Mexico City [60] and China [61].

### Model application

In this study, a domain with a horizontal resolution of 36 km covering China and surrounding countries in East Asia was employed [45]. The WRF v3.7.1 was utilized to produce meteorological inputs with initial and boundary conditions sourced from the FNL (Final) Operational Global Analysis Data National Center for Atmospheric Research, which is available on 1.0 × 1.0 degree grids continuously every six hours (http://dss.ucar.edu/datasets/ds083.2/). The outputs generated by WRF were then processed using the Meteorology-Chemistry Interface Processor (MCIP) v4.2 to generate inputs for CMAQ.

Anthropogenic emissions were derived from the MEICv1.3 developed by Tsinghua University (http://www.meicmodel.org) [62]. In this study, we compared the MEIC default emissions (Biogenic) with our newly developed combustion isoprene emissions described above as PKU (New). Wildfire emissions were obtained from the Fire Inventory from National Center for Atmospheric Research (FINN) [63]. A detailed comparison of these two emission inventories is listed in Table S4.

To estimate the biogenic emissions of China accurately, we customized EFs for each VOC category, plant functional type (PFT) and Leaf Area Index (LAI) and utilized the Moderate Resolution Imaging Spectroradiometer (MODIS) at a spatial resolution of 500 m as the required inputs for MEGAN v2.1 [5], with meteorological parameters provided by simulations from the WRF model. More detailed information can be found in Ma *et al.* (2022) [32]. Four simulation episodes (February, April, July and October) were chosen for the years 2000, 2005, 2010, 2015 and 2016 to analyze the seasonal and annual variations in isoprene-derived SOA.

To validate the model's performance, we compared our results with previous isoprene observation data and previous simulation results [31] at the Tongyu site in Northeast China from January 2013 to March 2013, as depicted in Fig. S15. In a study by Zhang *et al.* (2020) [31], the model exhibited a clear underestimation, particularly during wintertime, for sites in Northeast, Northwest and North China, although its predictions were much closer to the observations during the growing season [31]. The authors speculated that the increase in isoprene during wintertime might be linked to combustion sources. In our study, the hypothesis was further supported by the inclusion of combustion sources in our new isoprene emission inventory. After integrating the new inventory, our modeled isoprene (0.067 ppbv, mean) substantially exceeded that (0.003 ppbv, mean) modeled without the addition of combustion sources (Fig. S16). Although our modeled isoprene still underestimated the observations, it could explain 24% of the observed concentrations, compared to 1% in previous simulations without combustion isoprene sources. Additionally, the coarse grid resolution (36 × 36 km) may have contributed to the underestimation, as local combustion sources could result in higher isoprene mixing ratios at the observation site compared to the grid average.

Previous studies have shown that high isoprene levels in winter largely originate from combustion sources, and a portion of this isoprene formed SOA, as reported by Ding *et al.* (2016) [30]. Lower temperatures in winter increase the gas-particle partitioning coefficient, resulting in higher yields of isoprene-derived SOA [3]. Additionally, NOx levels significantly influence SOA yields; under low-NOx conditions, isoprene photo-oxidation forms IEPOX, while under high-NOx conditions, it forms MAE, as discussed by Wennberg *et al.* (2018) [47]. In our work, we enhanced the SOA formation pathways to simulate SOA formation through surface-controlled reactive uptake of dicarbonyls, IEPOX, MAE, and in-cloud processing of GLY and MGLY. Despite these improvements, some SOA formation pathways remain unexplained or incomplete, contributing to ongoing underestimation of SOA in our model.

## Supplementary Material

nwae474_Supplemental_File

## Data Availability

Data processing techniques are available on request from the corresponding author.
