# Supplementary material for: Combustion-related isoprene contributes substantially to the formation of wintertime secondary organic aerosols
Source: Natl Sci Rev. 2025 Jan 2;12(3):nwae474. doi: 10.1093/nsr/nwae474 (PMC11827594; doi:10.1093/nsr/nwae474)
Supplement: nwae474_Supplemental_File [file nwae474_supplemental_file.pdf]

Supporting information

Combustion-related isoprene contributes substantially to the formation of wintertime secondary organic aerosols

Yanli Zhang[1]†, Yatai Men[2]†, Hao Guo[1]†, Guofeng Shen[2,3,]*, Yang Gao[4], Rui Xiong[2], Shu Tao[2,3], Xinming Wang[1]*

[1]State Key Laboratory of Organic Geochemistry and Guangdong Key Laboratory of Environmental Protection and Resources Utilization, Guangzhou Institute of Geochemistry, Chinese Academy of Sciences, Guangzhou 510640, China.

[2]Laboratory for Earth Surface Processes, College of Urban and Environmental Sciences, Peking University, Beijing 100871, China

[3]Institute of Carbon Neutrality, Peking University, Beijing 100871, China

[4]Frontiers Science Center for Deep Ocean Multispheres and Earth System/Key Laboratory of Marine Environmental Science and Ecology, Ministry of Education, Ocean University of China, Qingdao 266100, China

*Corresponding author. Email: gfshen12@pku.edu.cn (G.S.); wangxm@gig.ac.cn (X.W.)

†These authors contributed equally to this work.

There are 1 text, 4 tables and 17 figures in this appendix.

Table S1. Annual emissions of isoprene from biogenic and combustion-related sources, including open biomass burning, biofuels and coal combustions in the residential sector in this study, in China from 2000 to 2016.

Year	Combustion Emissions (Gg)	Biogenic Emissions (Gg)	Total Isoprene Emissions (Gg)
2000	52.04	5871.59	5923.63
2001	48.91	6217.42	6266.33
2002	46.99	6301.51	6348.50
2003	48.46	6427.66	6476.11
2004	46.56	6706.45	6753.02
2005	43.69	6768.18	6811.87
2006	42.68	7129.93	7172.61
2007	40.26	7504.75	7545.01
2008	38.25	7811.42	7849.67
2009	35.89	7950.08	7985.97
2010	32.56	8158.05	8190.61
2011	30.18	9188.34	9218.52
2012	22.89	9776.95	9799.85
2013	24.48	10099.98	10124.45
2014	22.86	11250.44	11273.30
2015	22.45	12400.90	12423.35
2016	14.82	12597.56	12612.38

Table S2. Classification of combustion source activities and data sources

Category	Fuel/activity	Source
Forest Wildfires	Savanna, grassland, and shrubland fires	GFED4.1s
	Boreal forest fires	GFED4.1s
	Temperate forest fires	GFED4.1s
	Tropical deforestation & degradation	GFED4.1s
	Peat fires	GFED4.1s
Open-field Agricultural Residue Burning	Agricultural waste burning	GFED4.1s
Residential Coal Consumption	Anthracite	PKU-FUEL
	Coking coal	PKU-FUEL
	Bituminous coal	PKU-FUEL
	Lignite	PKU-FUEL
	Peat	PKU-FUEL
	Patent fuel	PKU-FUEL
	Honeycomb/Coal briquettes	PKU-FUEL
Residential Biomass Consumption	Indoor brush wood burning	PKU-FUEL
	Charcoal	PKU-FUEL
	Indoor crop residue burning	PKU-FUEL
	Indoor corncob burning	PKU-FUEL
	Indoor dung cake burning	PKU-FUEL
	Indoor brush wood burning	PKU-FUEL

Table S3. Results of document retrieval on isoprene emission factors from combustion source

Title	Journal	Publish Time	Combustion Source	Emission Factors (g/kg)
Emission of trace gases and aerosols from biomass burning–an updated assessment[1]	Atmospheric Chemistry and Physics	2019	Agricultural residues (open)	0.17
Emission of trace gases and aerosols from biomass burning[2]	Global biogeochemical cycles	2001	Biofuel burning	0.15
Emission of trace gases and aerosols from biomass burning–an updated assessment[1]	Atmospheric Chemistry and Physics	2019	Biofuels (without dung)	0.06
Emission factors for open and domestic biomass burning for use in atmospheric models[3]	Atmospheric Chemistry and Physics	2011	Boreal forest	0.15
Emission of trace gases and aerosols from biomass burning–an updated assessment[1]	Atmospheric Chemistry and Physics	2019	Boreal forest	0.074
Emission of trace gases and aerosols from biomass burning[2]	Global biogeochemical cycles	2001	Charcoal burning	0.017
Emission of trace gases and aerosols from biomass burning–an updated assessment[1]	Atmospheric Chemistry and Physics	2019	Charcoal burning	0.12
Open burning of rice, corn and wheat straws: primary emissions, photochemical aging, and secondary organic aerosol formation[4]	Atmospheric Chemistry and Physics	2017	corn straw	0.026
Emission factors for open and domestic biomass burning for use in atmospheric models[3]	Atmospheric Chemistry and Physics	2011	Crop residue	0.38
Emission factors of atmospheric and climatic pollutants from crop residues burning[5]	Journal of the Air & Waste Management Association	2018	crop residues	0.19
Emission of trace gases and aerosols from biomass burning–an updated assessment[1]	Atmospheric Chemistry and Physics	2019	Dung burning	0.2
Emission factors for open and domestic biomass burning for use in atmospheric models[3]	Atmospheric Chemistry and Physics	2011	Extratropical forest	0.15

Title	Journal	Publish Time	Combustion Source	Emission Factors (g/kg)
Emission of trace gases and aerosols from biomass burning[2]	Global biogeochemical cycles	2001	Extratropical forest	0.1
Emission of trace gases and aerosols from biomass burning–an updated assessment[1]	Atmospheric Chemistry and Physics	2019	Firewood	0.001808
Air pollutant emissions and mitigation potential through the adoption of semi-coke coals and improved heating stoves: Field evaluation of a pilot intervention program in rural China[6]	Environmental Pollution	2018	Flaming Coal	0.0257
Boreal forest fire emissions in fresh Canadian smoke plumes: C 1-C 10 volatile organic compounds (VOCs), CO_2, CO, NO_2, NO, HCN and CH_3CN[7]	Atmospheric Chemistry and Physics	2011	Forest fire smoke plumes	0.074
Emissions of trace organic gases from Western US wildfires based on WE-CAN aircraft measurements[8]	Journal of Geophysical Research: Atmospheres	2021	Forest Wildfires (pine, fir, and spruce trees)	0.082
VOC emissions of smouldering combustion from Mediterranean wildfires in central Portugal[9]	Atmospheric Environment	2013	Forests wildfires	0.207
Emissions of trace gases from Australian temperate forest fires: emission factors and dependence on modified combustion efficiency[10]	Atmospheric Chemistry and Physics	2018	Forests wildfires	0.5
Emission of trace gases and aerosols from biomass burning–an updated assessment[1]	Atmospheric Chemistry and Physics	2019	Garbage burning	0.1
Emission of trace gases and aerosols from biomass burning–an updated assessment[1]	Atmospheric Chemistry and Physics	2019	Honeycomb briquette	0.000610592
Volatile organic compounds emissions from traditional and clean domestic heating appliances in Guanzhong Plain, China: Emission factors, source profiles, and effects on regional air quality[11]	Environment international	2019	Improved coal stove	0.00104
Emission of trace gases and aerosols from biomass burning–an updated assessment[1]	Atmospheric Chemistry and Physics	2019	Lump coal	0.00254966

Title	Journal	Publish Time	Combustion Source	Emission Factors (g/kg)
Emission factors for open and domestic biomass burning for use in atmospheric models[3]	Atmospheric Chemistry and Physics	2011	Pasture Maintenance	0.12
Emission of trace gases and aerosols from biomass burning–an updated assessment[1]	Atmospheric Chemistry and Physics	2019	Peat fires	0.52
Emission factors for open and domestic biomass burning for use in atmospheric models[3]	Atmospheric Chemistry and Physics	2011	Peatland	1.07
Open burning of rice, corn and wheat straws: primary emissions, photochemical aging, and secondary organic aerosol formation[4]		2017	rice straw	0.096
Emission factors for open and domestic biomass burning for use in atmospheric models[3]	Atmospheric Chemistry and Physics	2011	Savanna	0.039
Emission of trace gases and aerosols from biomass burning[2]	Global biogeochemical cycles	2001	Savanna and grassland	0.02
Emission of trace gases and aerosols from biomass burning–an updated assessment[1]	Atmospheric Chemistry and Physics	2019	Savanna and grassland	0.101
Volatile organic compounds emissions from traditional and clean domestic heating appliances in Guanzhong Plain, China: Emission factors, source profiles, and effects on regional air quality[11]	Environment international	2019	Semi-gasifier stove	0.00009
Air pollutant emissions and mitigation potential through the adoption of semi-coke coals and improved heating stoves: Field evaluation of a pilot intervention program in rural China[6]	Environmental Pollution	2018	Smoldering Coal	0.018
Emission of trace gases and aerosols from biomass burning[2]	Global biogeochemical cycles	2001	straw	0.00748858
Sugarcane burning emissions: Characterization and emission factors[12]	Atmospheric Environment	2018	Sugercane burning	0.19
Emission of trace gases and aerosols from biomass burning–an updated assessment[1]	Atmospheric Chemistry and Physics	2019	Temperate forest	0.1
Volatile organic compounds emissions from traditional and clean domestic heating	Environment international	2019	Traditional coal stove	0.029

Title	Journal	Publish Time	Combustion Source	Emission Factors (g/kg)
appliances in Guanzhong Plain, China: Emission factors, source profiles, and effects on regional air quality[11]				
Emission factors for open and domestic biomass burning for use in atmospheric models[3]	Atmospheric Chemistry and Physics	2011	Tropical forest	0.13
Emission of trace gases and aerosols from biomass burning[2]	Global biogeochemical cycles	2001	Tropical forest	0.016
Emission of trace gases and aerosols from biomass burning–an updated assessment[1]	Atmospheric Chemistry and Physics	2019	Tropical forest	0.22
Open burning of rice, corn and wheat straws: primary emissions, photochemical aging, and secondary organic aerosol formation[4]	Atmospheric Chemistry and Physics	2017	Wheat straw	0.06

Table S4. Comparison of the emission inventory used in the two scenarios.

Inventory Type	New inventory	Biogenic inventory
Anthropogenic	Multi-resolution Emission Inventory for China (MEICv1.3)	Multi-resolution Emission Inventory for China (MEICv1.3)
	Combustion isoprene emissions inventory PKU (New)	\
	including:	
	Forest Wildfires	\
	Open-field Agricultural Residue Burning	\
	Residential Coal Consumption	\
	Residential Biomass Consumption	\
Biogenic	Model for Emissions of Gases and Aerosols from Nature (MEGAN) v2.1	Model for Emissions of Gases and Aerosols from Nature (MEGAN) v2.1

Text S1:

Literature search of available studies on isoprene emission factors

This study conducted a systematic review of emission factors for isoprene from combustion sources. We performed a search in the Web of Science database using topic-specific terminology. The search theme terms were: TOPIC= (isoprene emission OR isoprene emission factor OR isoprene EF) AND ((coal OR wood OR biomass OR straws OR agricultural waste OR wood OR waste OR solid fuels OR residential fuels) OR ((fires OR wildfires) AND (Savanna OR grassland OR shrubland OR boreal forest OR peat OR agricultural waste OR tropical deforestation OR temperate forest))). After removing duplicates, we reviewed the titles and abstracts based on the inclusion criteria for full-text screening, excluding studies that did not meet these criteria. The inclusion criteria were: (1) availability of emission factor data for isoprene from combustion sources; (2) clear identification of the combustion source; and (3) adequate quality control information. The final studies included in the review are listed in Table S3.

Emission factors for isoprene were categorized by combustion source type, and each source's emission factors were assumed to follow a normal distribution. We calculated the mean and standard deviation of these values based on data from previous studies. This approach allowed us to quantify uncertainties in the emission factors, accounting for variations in measurement techniques, combustion conditions, and regional differences. These refinements aim to provide a more comprehensive and accurate representation of isoprene emissions and better reflect the uncertainty inherent in these estimates.

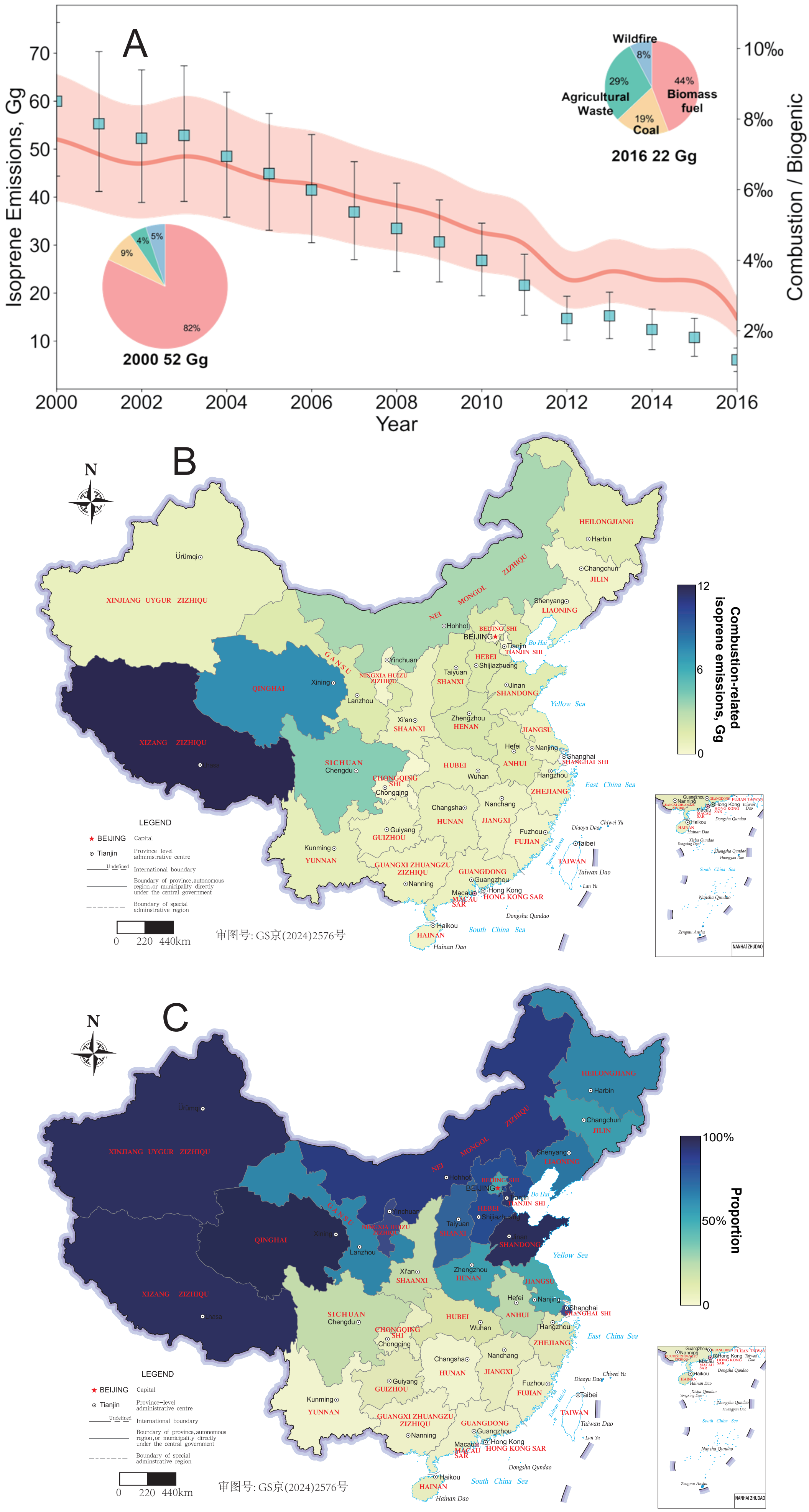

Figure S1. (A) Annual emissions of combustion-related isoprene (line), its proportion to the biogenic emission (square), and source profile of combustion-related emissions (pie). And the combustion-related isoprene emissions in 2000 (B) and 2016 (C).Data from Hong Kong, Macao and Taiwan are not available in this study.

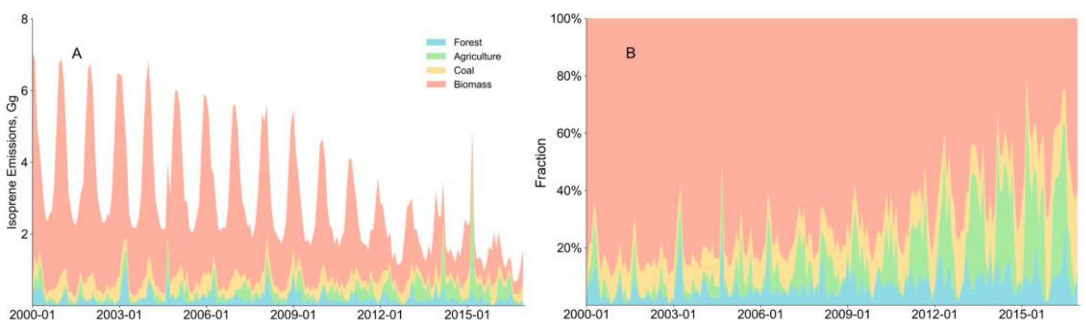

Figure S2. Trend of absolute values(A) and relative proportions(B) of combustion-related isoprene emissions from differentcombustion sources.

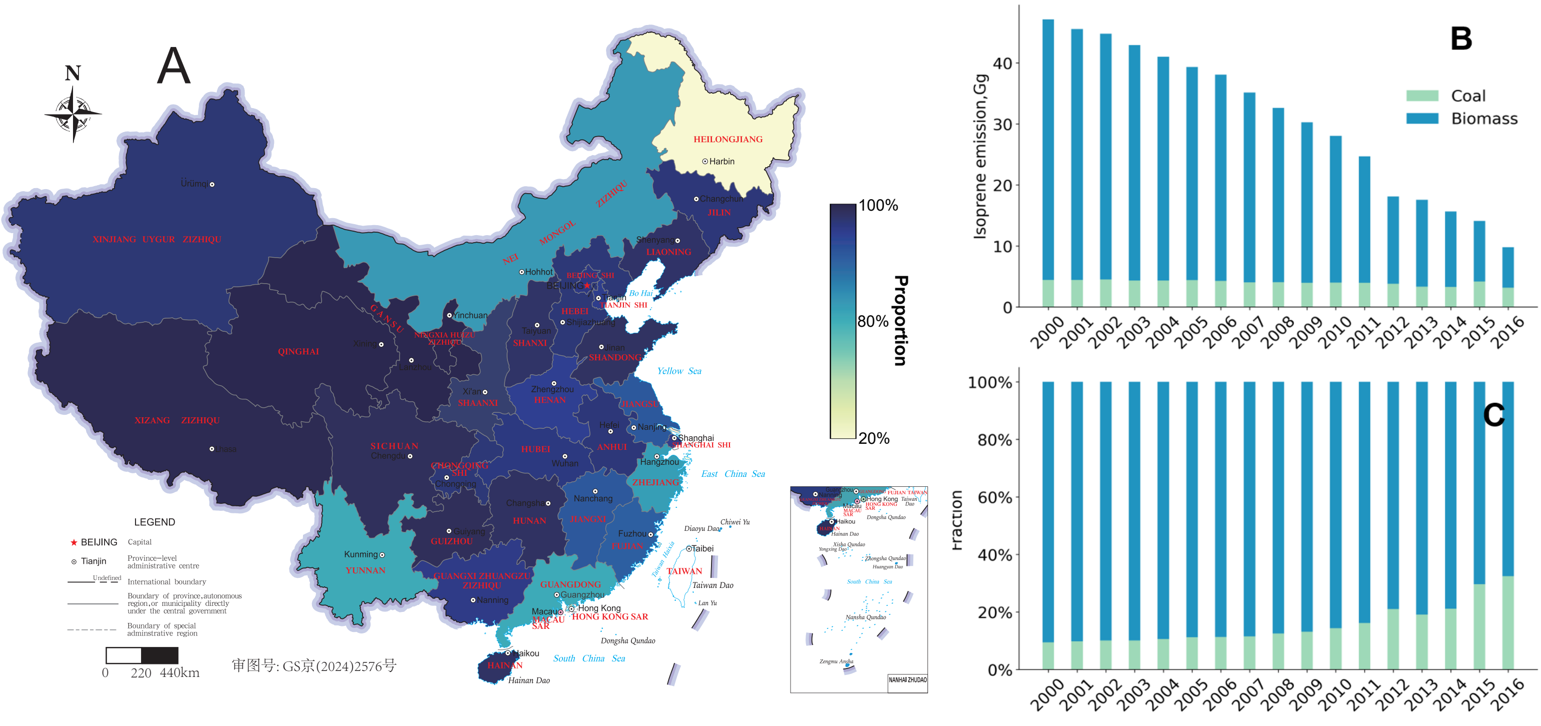

Figure S3. (A) Proportion of isoprene emissions from residential sources to combustion sources in 2000. And absolute value (B) and relative proportion (C) of isoprene emissions from coal and biomass combustion in the residential sector. Data from Hong Kong, Macao and Taiwan are not available in this study.

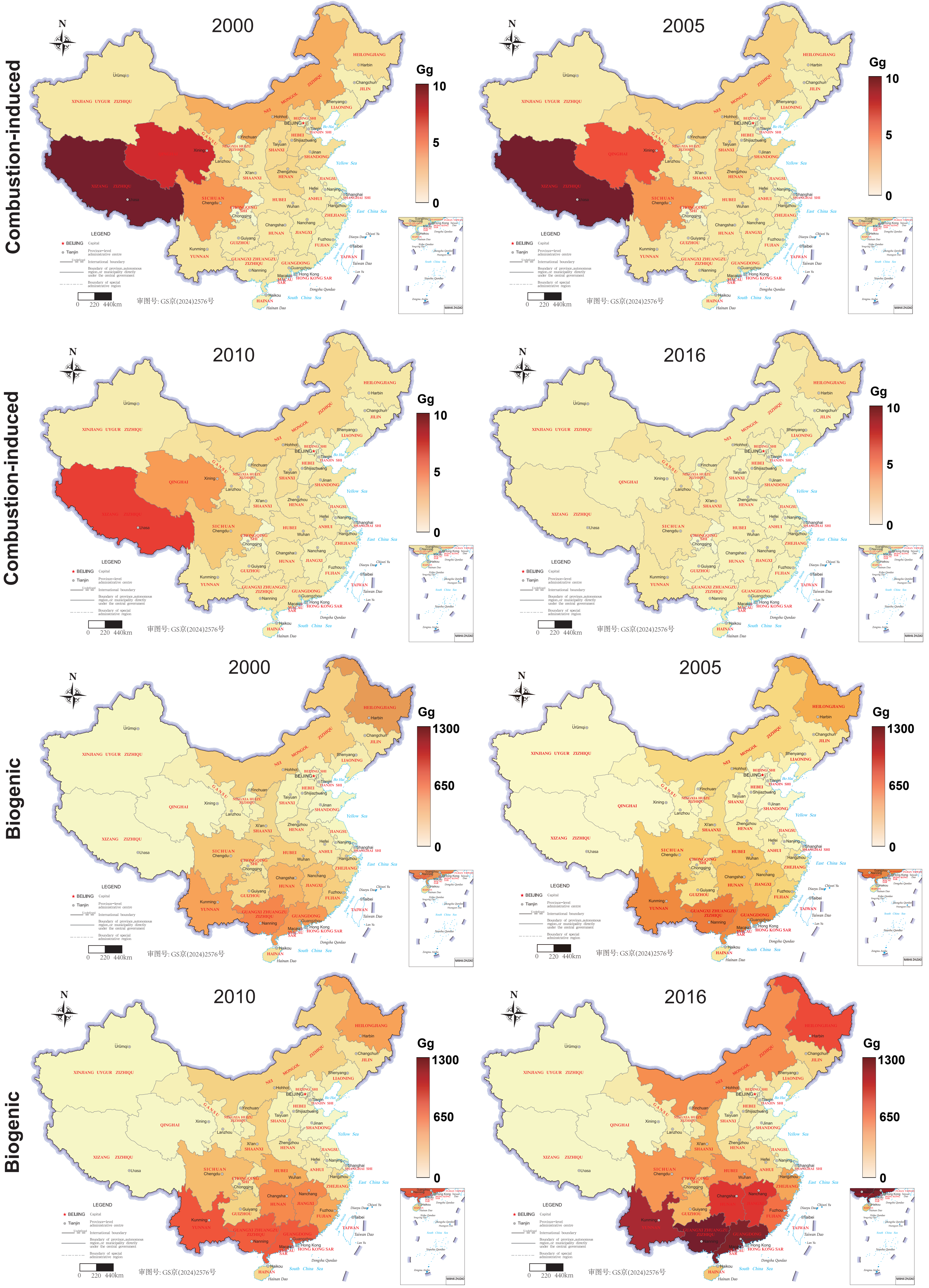

Figure S4. Spatial distribution of combustion-induced isoprene and biogenic isoprene in different years. Data from Hong Kong, Macao and Taiwan are not available in this study.

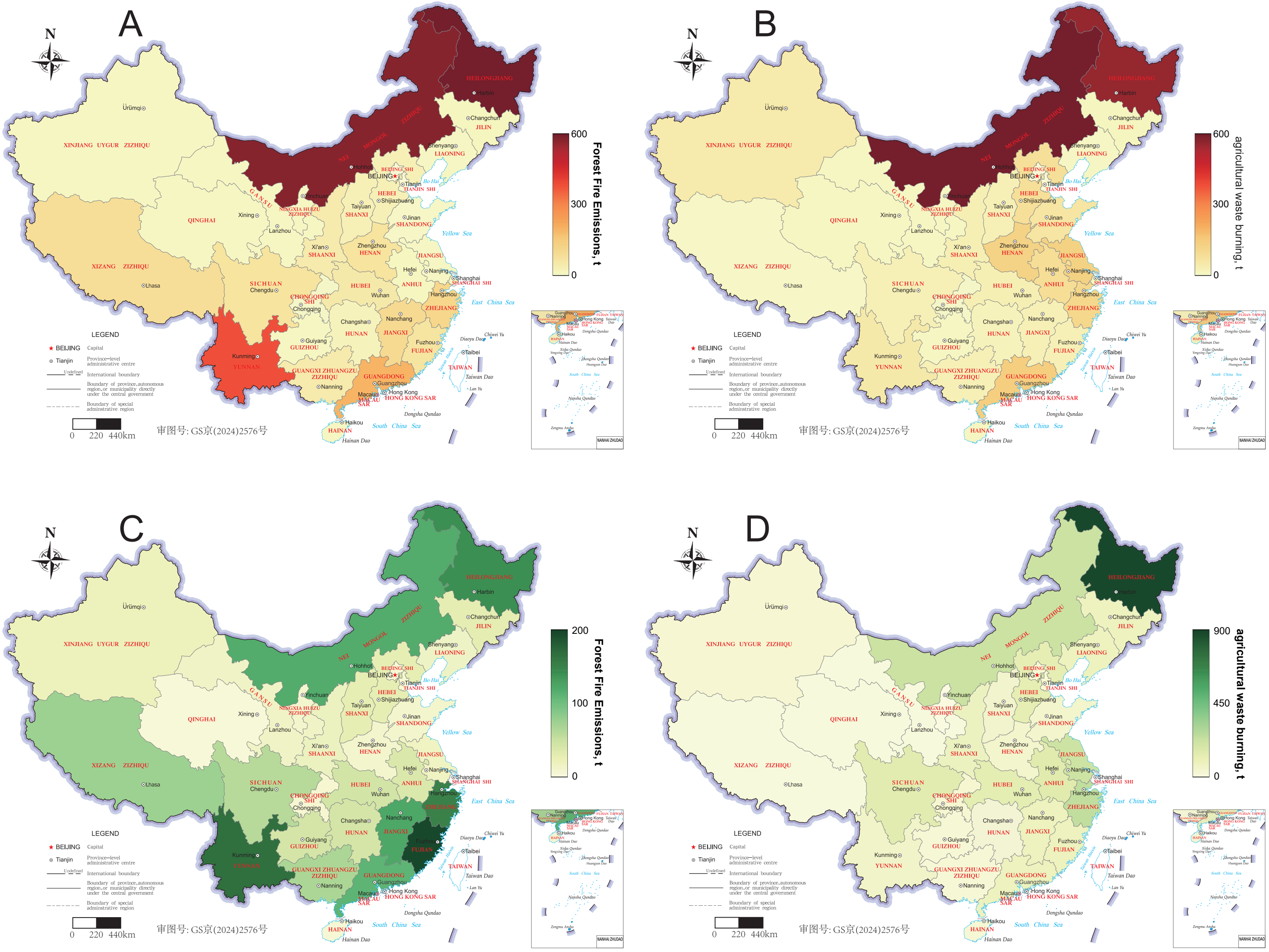

Figure S5. Isoprene emissions from forest fires and open-air agricultural waste burning in 2000 (A & B) and 2016 (C & D). Data from Hong Kong, Macao and Taiwan are not available in this study.

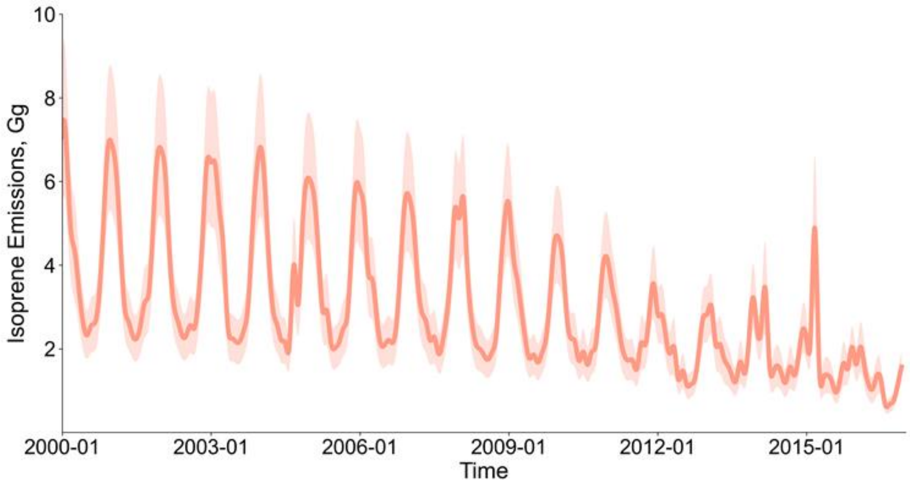

Figure S6. Monthly emissions of combustion-induced isoprene.

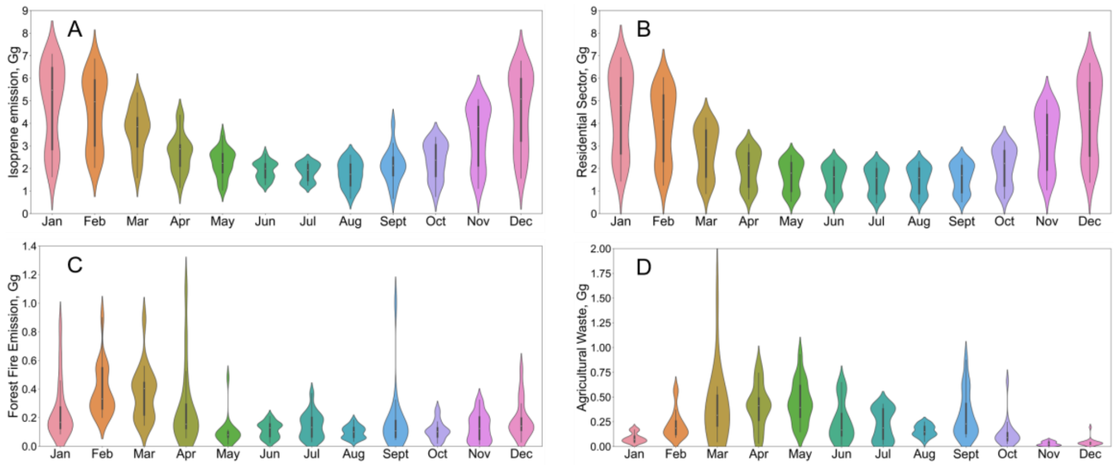

Figure S7. Monthly variations in isoprene emissions from thecombustion-relatedsources (A), residential-related combustion sources (B), forest wildfire (C), and agricultural waste combustion (D).

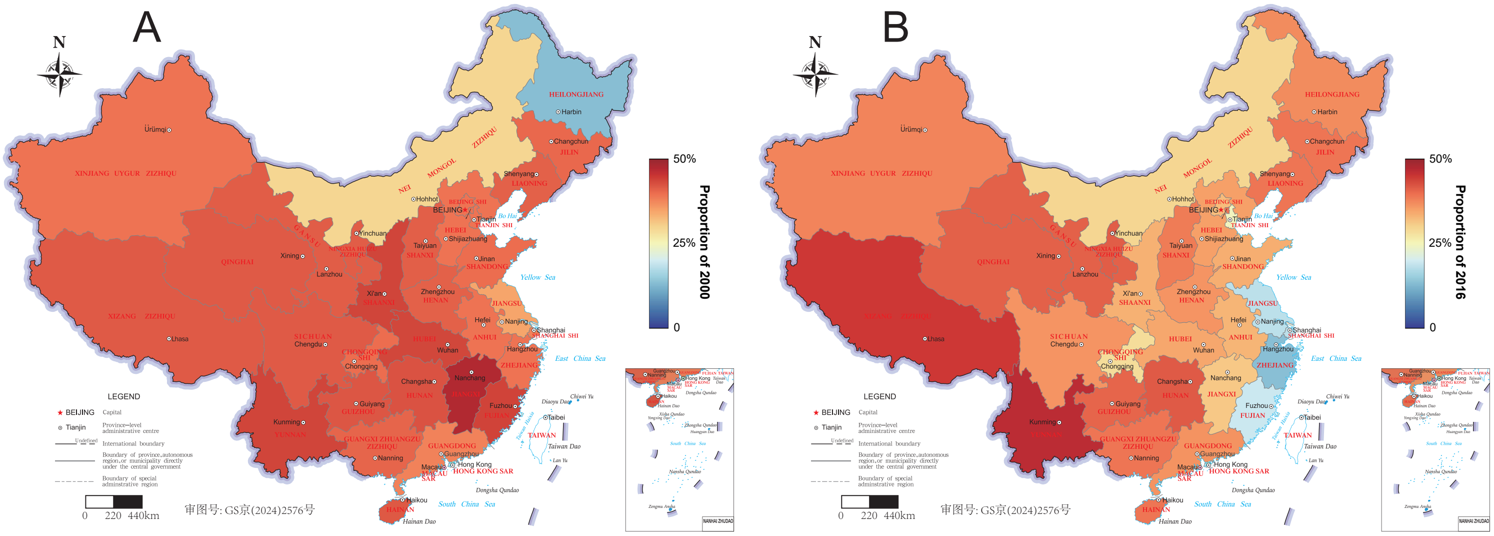

Figure S8. The proportion of wintertime combustion source emissions to the annual combustion source emissions in 2000 (A) and 2016 (B). Data from Hong Kong, Macao and Taiwan are not available in this study.

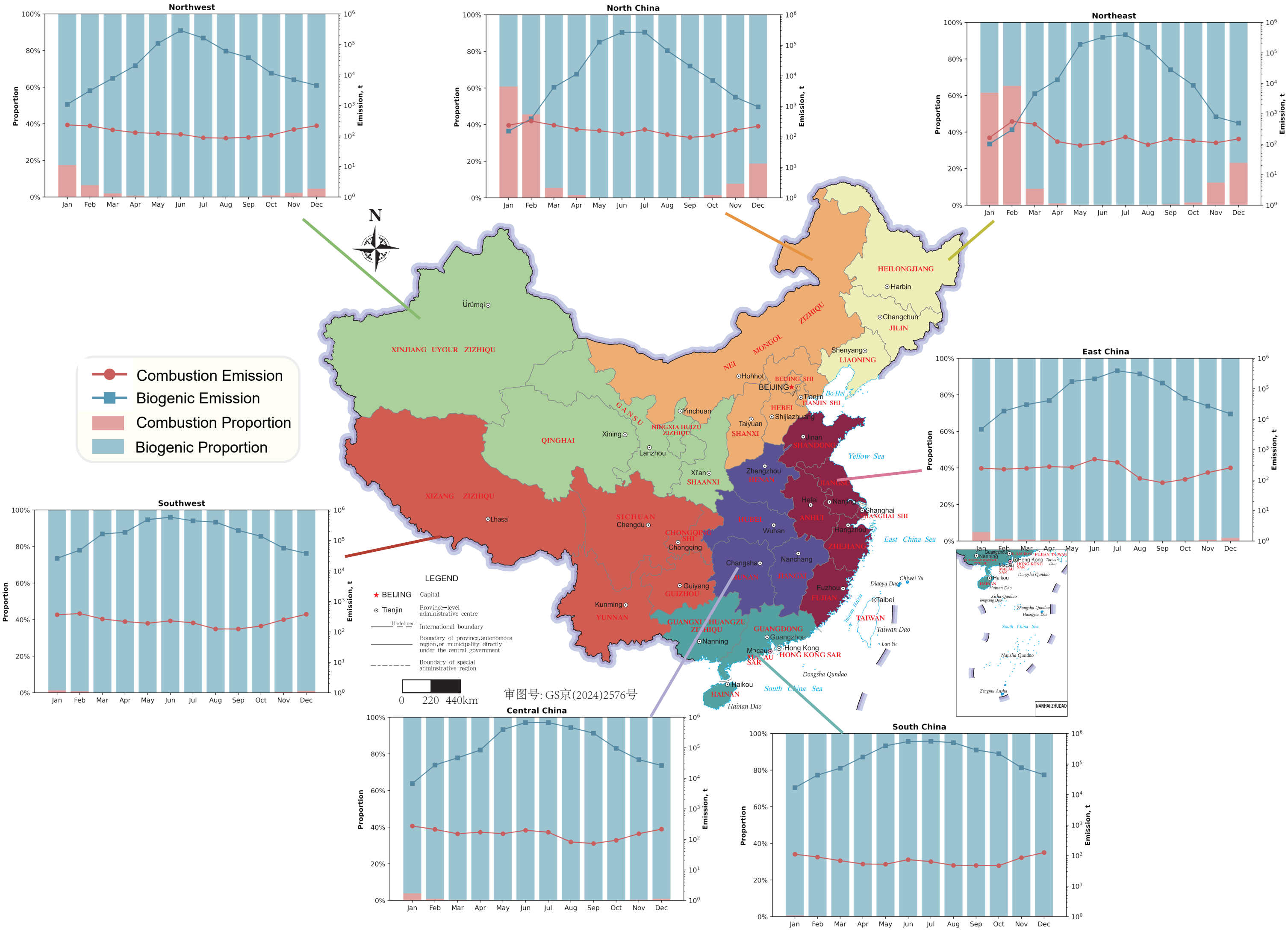

Figure S9. Monthly changes in isoprene emissions and their proportion to total emissions related to biological and combustion sources in different regions of China in 2016. Data from Hong Kong, Macao and Taiwan are not available in this study.

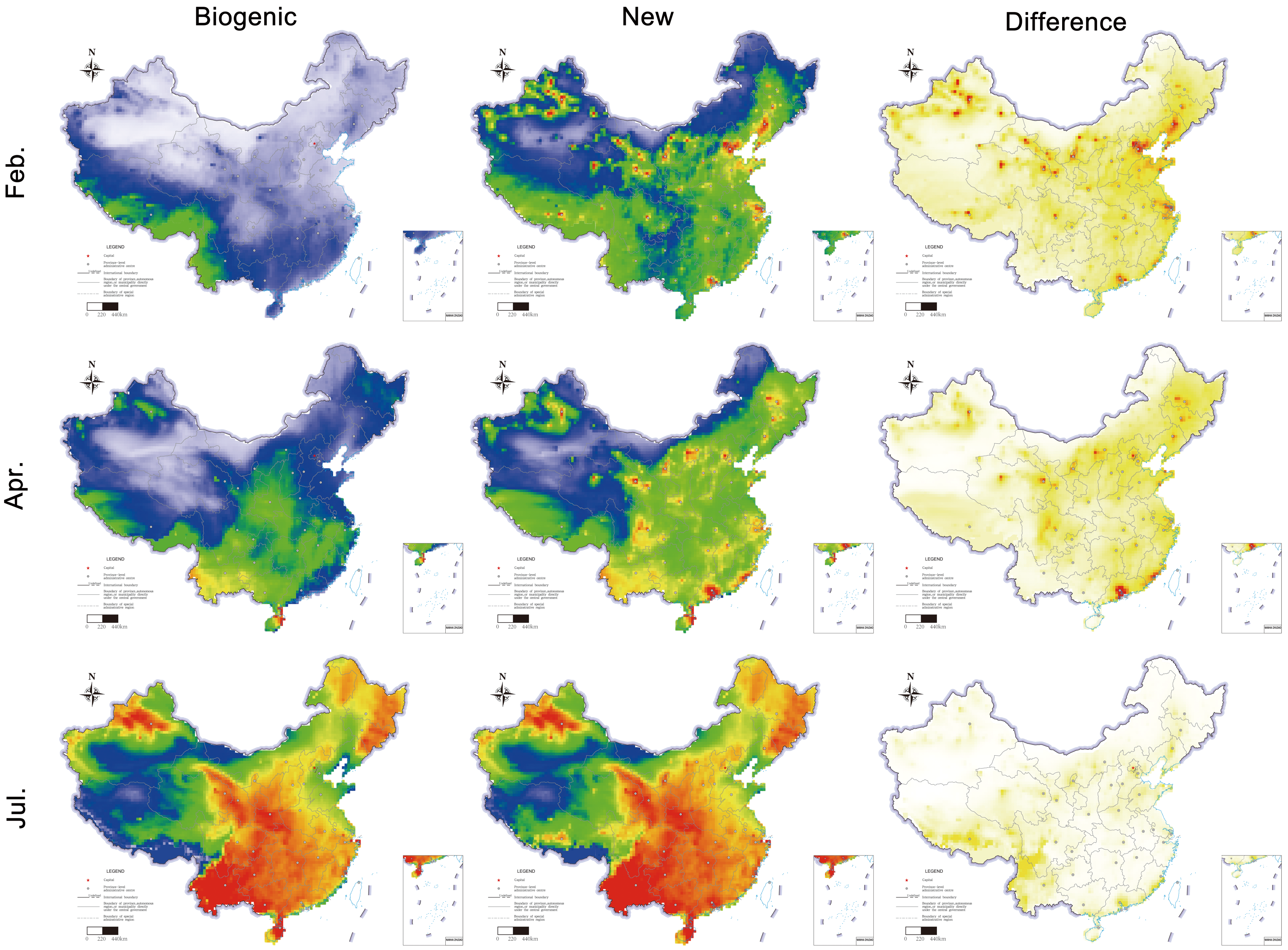

审图号：GS京(2024)2576号

Figure S10. Seasonal variations of SOA formed by isoprene emissions' proportions in total SOA in Biogenic, New inventories and their difference. Data from Hong Kong, Macao and Taiwan are not available in this study.

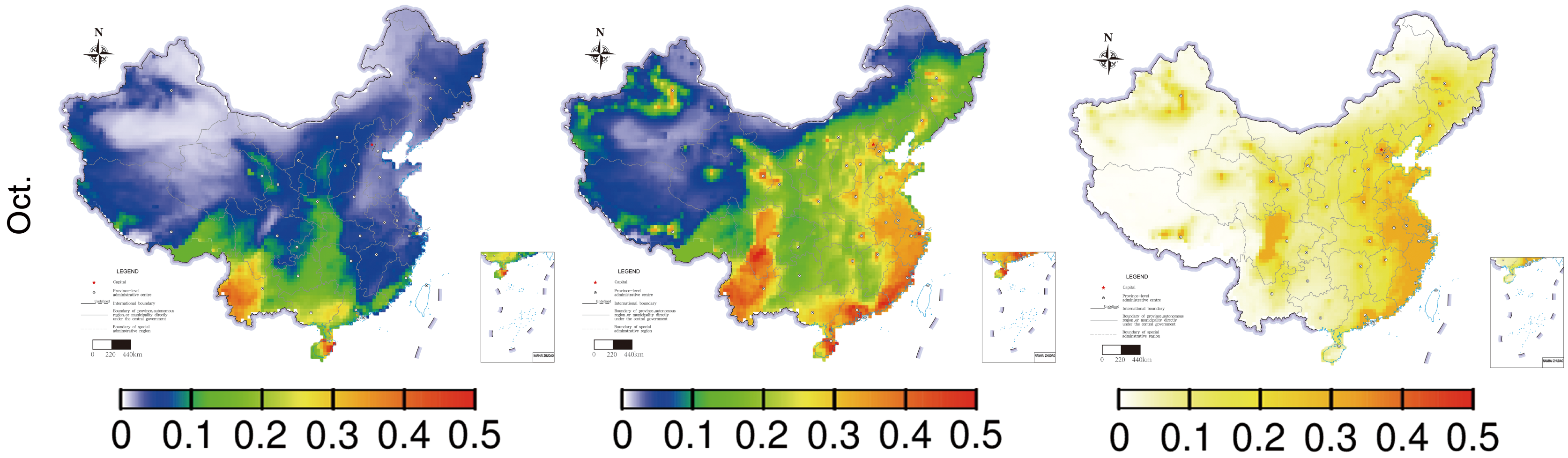

审图号：GS京(2024)2576号

Continue Figure S10. Seasonal variations of SOA formed by isoprene emissions' proportions in total SOA in Biogenic, New inventories and their difference. Data from Hong Kong, Macao and Taiwan are not available in this study.

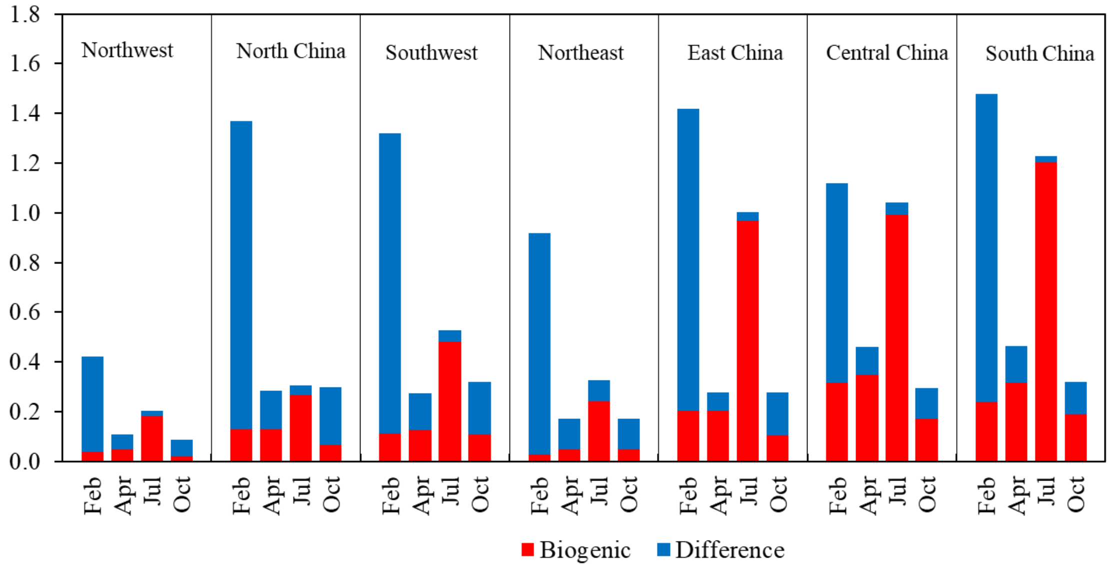

Figure S11. Seasonal variations of isoprene-derived SOA(μg/m3) in simulations using the Biogenic isoprene emission inventory (red) and the differences between the Biogenic and New emission inventories (blue) at seven China regions in 2016.

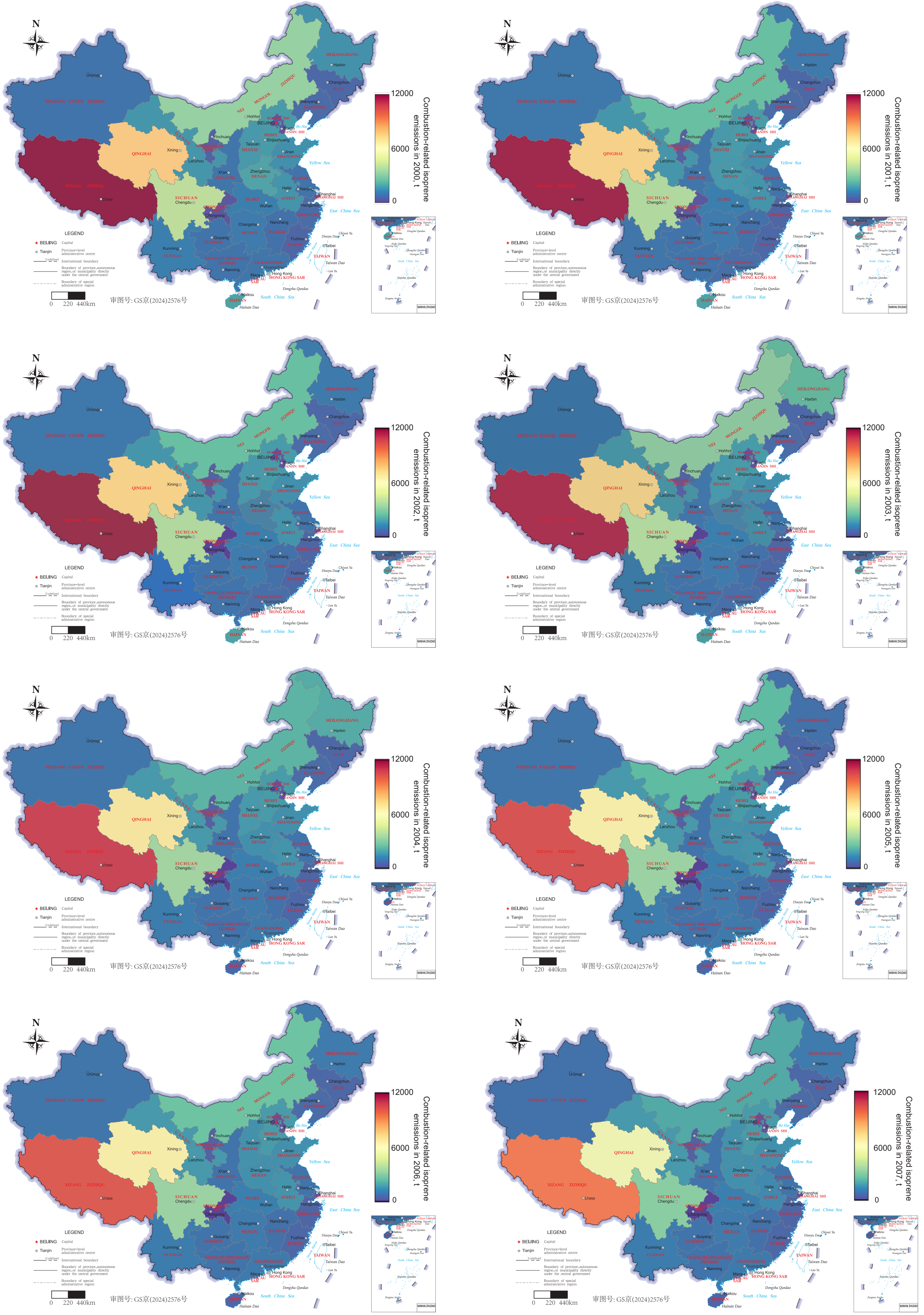

Figure S12. Annual emissions of isoprene related to combustion sources from 2000 to 2016. Data from Hong Kong, Macao and Taiwan are not available in this study.

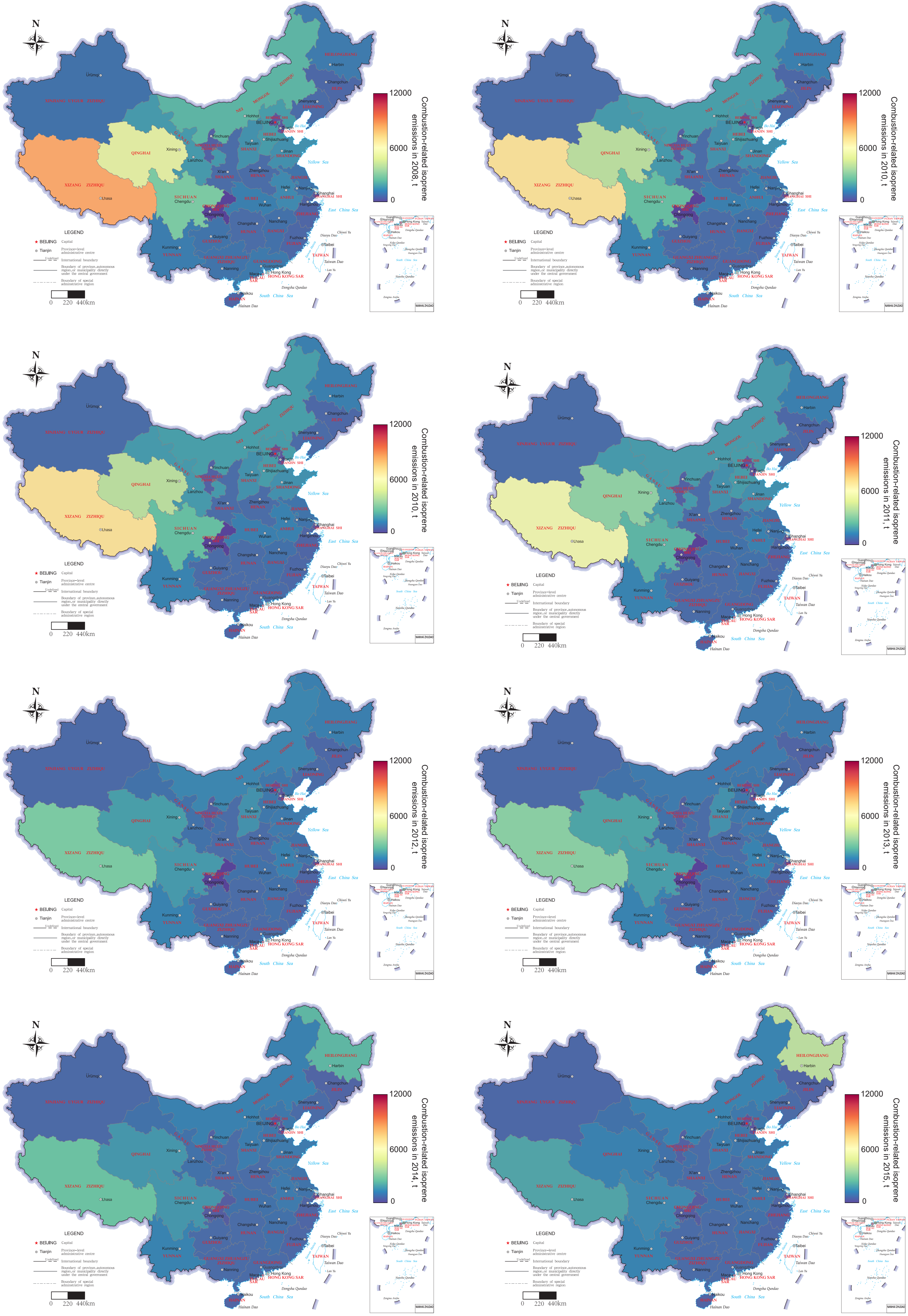

Continue Figure S12. Annual emissions of isoprene related to combustion sources from 2000 to 2016. Data from Hong Kong, Macao and Taiwan are not available in this study.

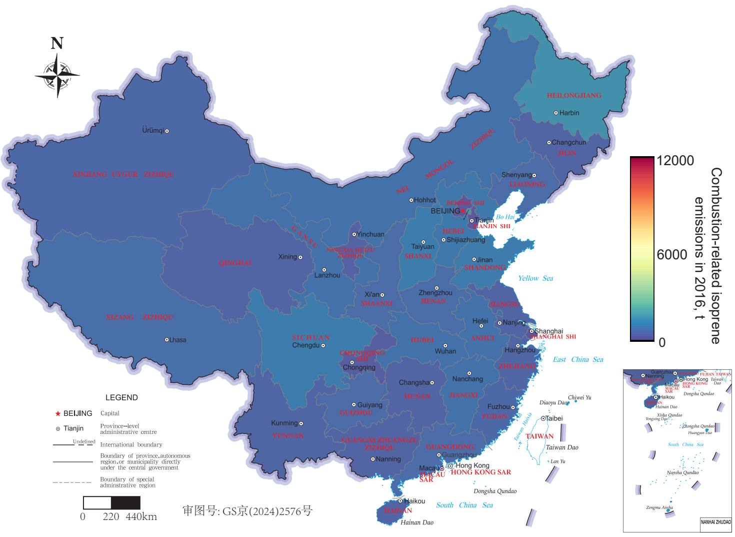

Continue Figure S12. Annual emissions of isoprene related to combustion sources from 2000 to 2016. Data from Hong Kong, Macao and Taiwan are not available in this study.

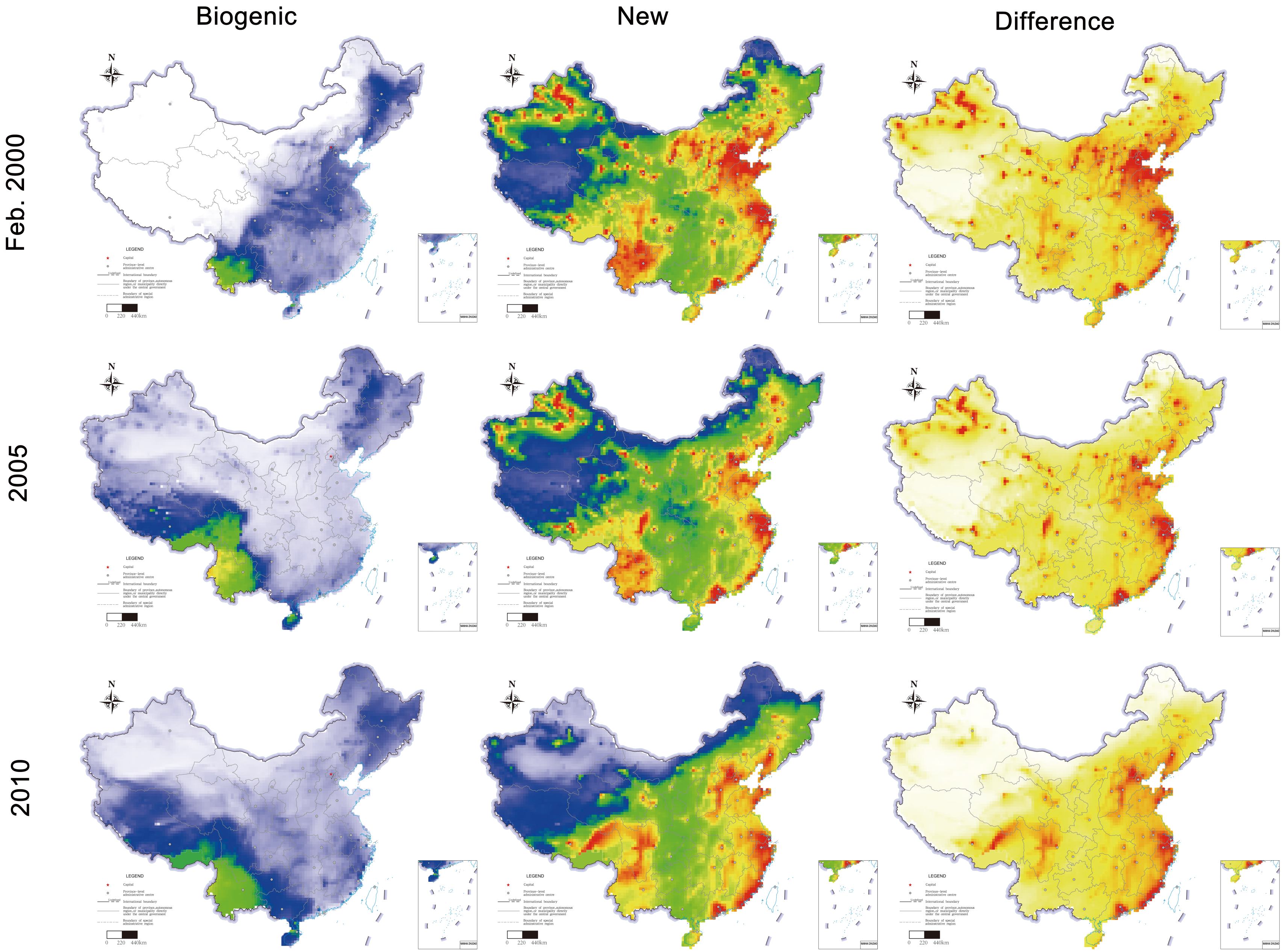

审图号：GS京(2024)2576号

Figure S13. Historical variations of SOA formed by isoprene emissions' proportions in total SOA in Biogenic, New inventories and their difference. Data from Hong Kong, Macao and Taiwan are not available in this study.

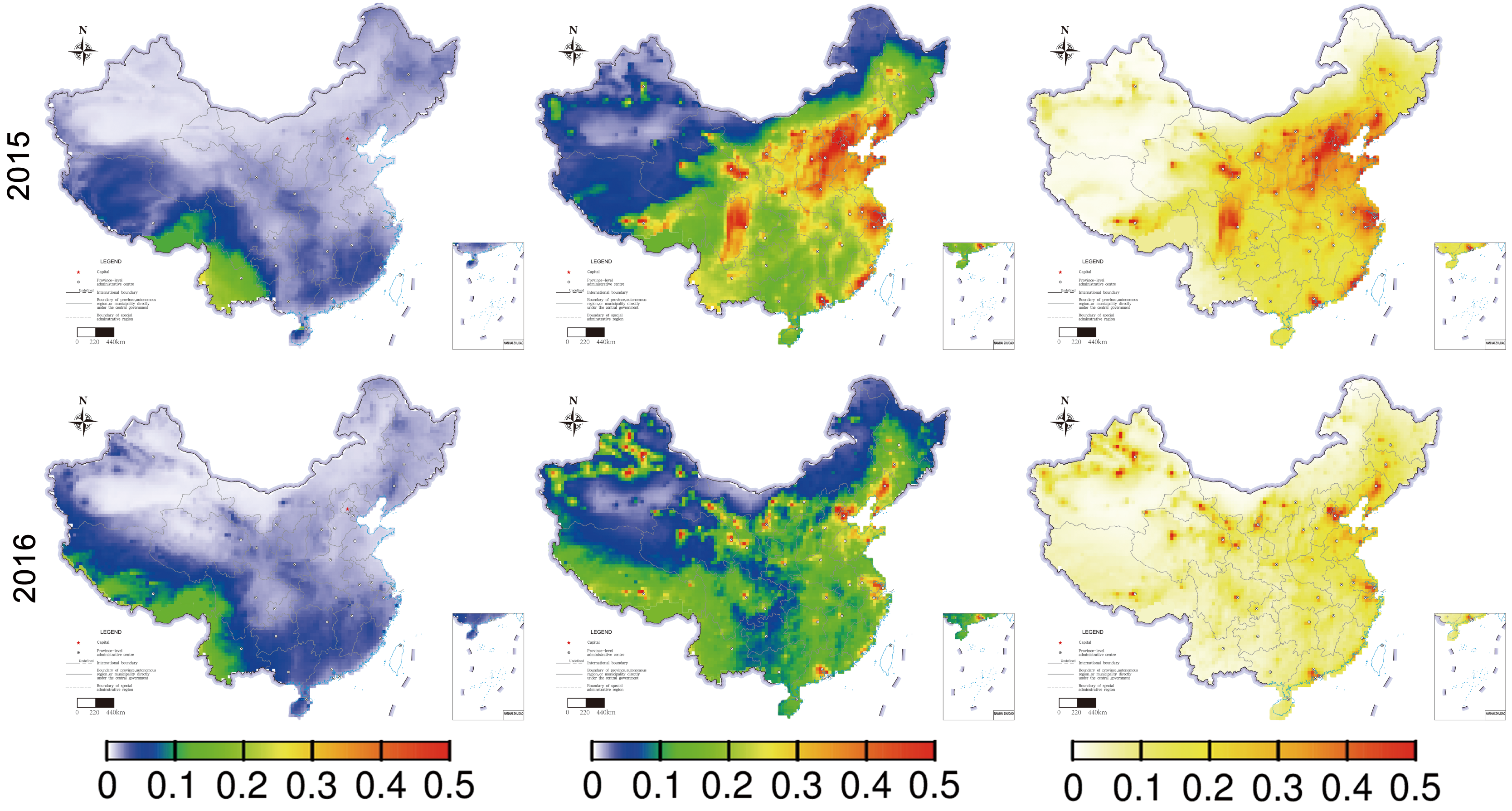

审图号：GS京(2024)2576号

Continue Figure S13. Historical variations of SOA formed by isoprene emissions' proportions in total SOA in Biogenic, New inventories and their difference. Data from Hong Kong, Macao and Taiwan are not available in this study.

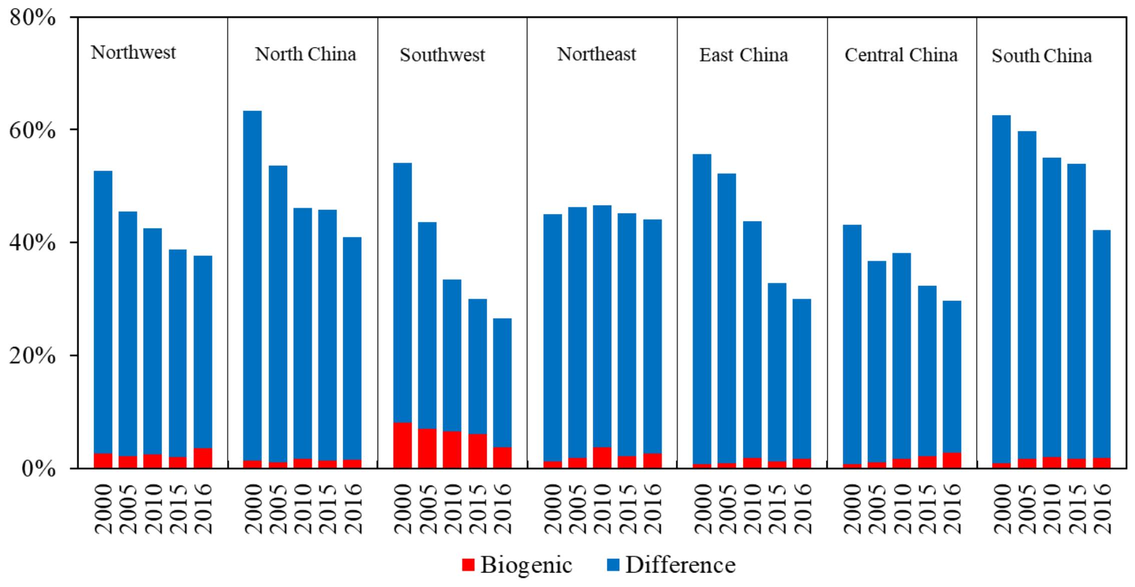

Figure S14. Proportions of isoprene-derived SOAin total SOAat 7 major regions in winter f rom 2000 to 2016 in Biogenic inventory(red) and the simulation differences(blue) between the Biogenic and New inventories at 7 China regions in 2016.

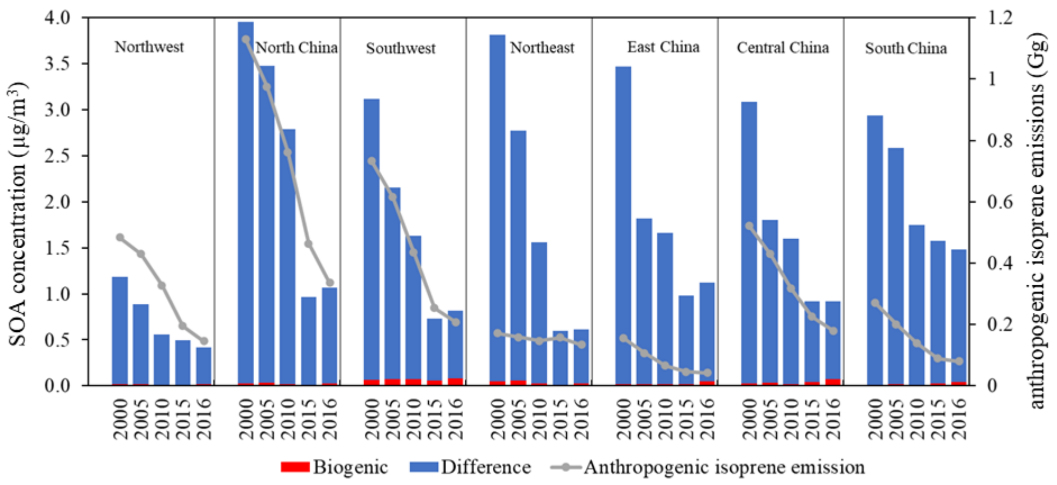

Figure S15. Annual variations of SOA concentration (µg/m3) at wintertime with total anthropogenic isoprene emissions (Gg, y axis on right) at 7 major regions in Biogenic inventory (red) and the simulation differences (blue) between the Biogenic and New inventories at 7 China regions in 2016.

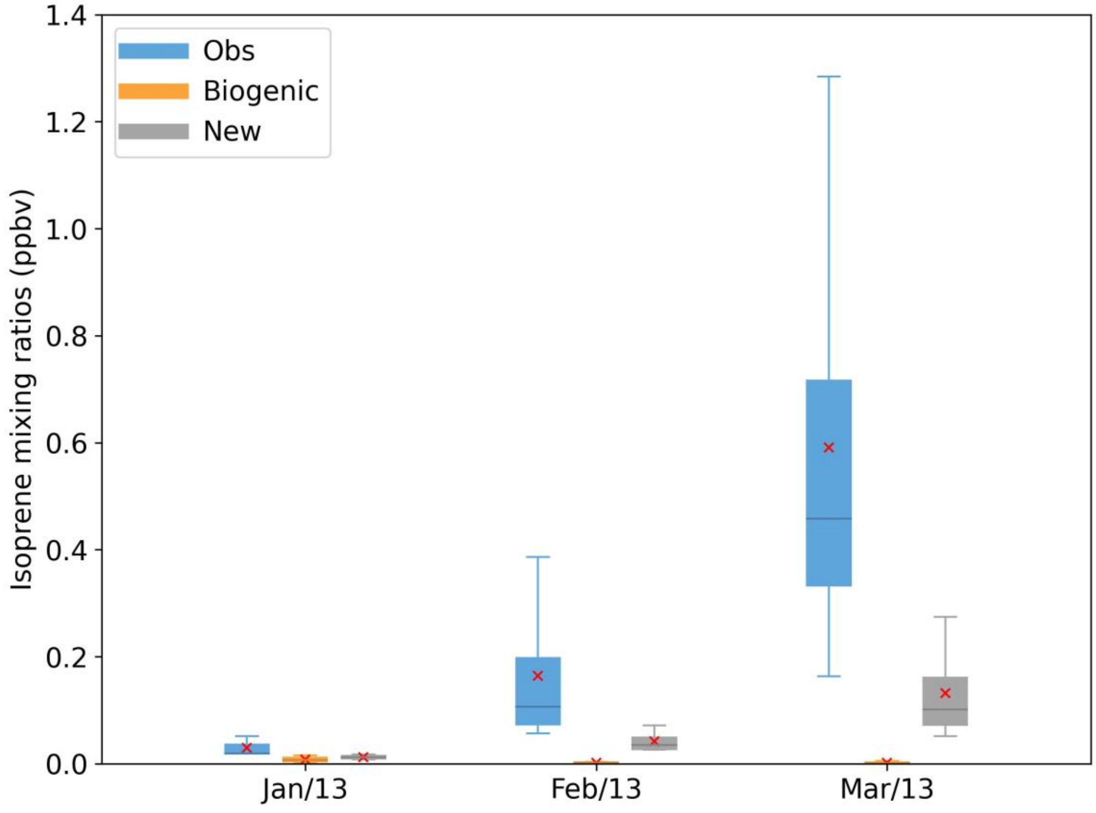

Figure S16. Comparison of monthly variations of isoprene mixing ratios(ppbv) between Biogenic and Newinventories, previous model simulation in Zhang et al, 2020 and ambient observation at Tongyusite from January 2013 to March 2013[13].

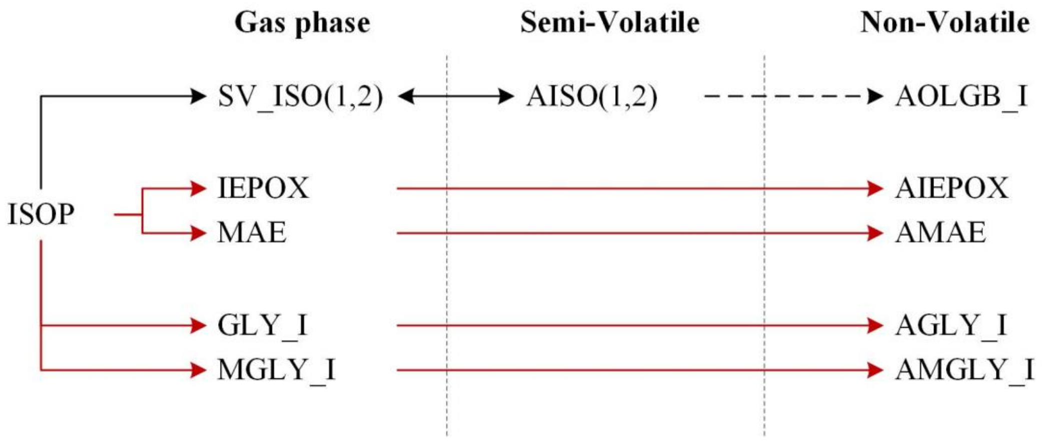

Figure S17. A conceptual figure of isoprene-derived SOA formation pathway in updated chemical mechanism of CMAQ.

References

1. Andreae MO. Emission of trace gases and aerosols from biomass burning–an updated assessment. *Atmos Chem Phys* 2019; **19(13)**:8523-8546.
2. Andreae MO, Merlet P. Emission of trace gases and aerosols from biomass burning. *Glob Biogeochem Cycles* 2001; **15(4)**:955-966.
3. Akagi S, Yokelson RJ, Wiedinmyer C, et al. Emission factors for open and domestic biomass burning for use in atmospheric models. *Atmos Chem Phys* 2011; **11(9)**:4039-4072.
4. Fang Z, Deng W, Zhang Y, et al. Open burning of rice, corn and wheat straws: primary emissions, photochemical aging, and secondary organic aerosol formation. *Atmos Chem Phys* 2017; **17(24)**:14821-14839.
5. Santiago-De La Rosa N, González-Cardoso G, Figueroa-Lara JdJ, et al. Emission factors of atmospheric and climatic pollutants from crop residues burning. *J Air Waste Manag Assoc* 2018; **68(8)**:849-865.
6. Liu Y, Zhang Y, Li C, et al. Air pollutant emissions and mitigation potential through the adoption of semi-coke coals and improved heating stoves: Field evaluation of a pilot intervention program in rural China. *Environ Pollut* 2018; **240**:661-669.
7. Simpson IJ, Akagi S, Barletta B, et al. Boreal forest fire emissions in fresh Canadian smoke plumes: C 1-C 10 volatile organic compounds (VOCs), CO2, CO, NO2, NO, HCN and CH3CN. *Atmos Chem Phys* 2011; **11(13)**:6445-6463.
8. Permar W, Wang Q, Selimovic V, et al. Emissions of trace organic gases from Western US wildfires based on WE-CAN aircraft measurements. *J Geophys Res Atmos* 2021; **126(11)**:e2020JD033838.
9. Evtyugina M, Calvo AI, Nunes T, et al. VOC emissions of smouldering combustion from Mediterranean wildfires in central Portugal. *Atmos Environ* 2013; **64**:339-348.
10. Guérette E-A, Paton-Walsh C, Desservettaz M, et al. Emissions of trace gases from Australian temperate forest fires: emission factors and dependence on modified combustion efficiency. *Atmos Chem Phys* 2018; **18(5)**:3717-3735.
11. Sun J, Shen Z, Zhang L, et al. Volatile organic compounds emissions from traditional and clean domestic heating appliances in Guanzhong Plain, China: Emission factors, source profiles, and effects on regional air quality. *Environ Int* 2019; **133**:105252.
12. Mugica-Álvarez V, Hernández-Rosas F, Magaña-Reyes M, et al. Sugarcane burning emissions: Characterization and emission factors. *Atmos Environ* 2018; **193**:262-272.
13. Zhang Y, Zhang R, Yu J, et al. Isoprene mixing ratios measured at twenty sites in China during 2012–2014: Comparison with model simulation. *J Geophys Res Atmos* 2020; **125(24)**:e2020JD033523.
